# Sleeping giants: temporal, seasonal, and spatial variations in the 24-h activity budget of *Hippopotamus amphibius*

**DOI:** 10.1093/jmammal/gyaf068

**Published:** 2025-09-19

**Authors:** Victoria L Inman, Keith E A Leggett

**Affiliations:** Centre for Ecosystem Science, School of Biological, Earth and Environmental Sciences, UNSW Sydney, Kensington, NSW 2052, Australia; Centre for Ecosystem Science, School of Biological, Earth and Environmental Sciences, UNSW Sydney, Kensington, NSW 2052, Australia

**Keywords:** activity budget, behavior, Botswana, Hippopotamus, nocturnal

## Abstract

Understanding animal activity budgets is essential for assessing habitat use and ecological roles, with important implications for conservation. Despite their ecological significance, the behavior of Common Hippopotamus (*Hippopotamus amphibius*) remains poorly studied, particularly at night. This study aimed to (i) quantify the 24-h activity budget of hippos; (ii) evaluate how behavior changed over the day and varied seasonally; (iii) examine how behavior varied between different areas; and (iv) between age classes. This study presents the first 24-h observational activity budget of hippos and the first behavioral data from Botswana. Hippo behavior varied significantly by time of day, season, study area, and age class. Hippos exhibited a well-defined circadian rhythm, with activity peaking and dipping at sunrise, sunset, around midday, and midnight. Contrary to the persistent assumption that hippos rest in water by day and graze on land all night, hippos in this study fed during only a quarter of the night and were active for a similar proportion of the day. Hippos often spent hours of the day on land feeding or basking in the sun, challenging the idea that they rely heavily on water to prevent their skin from cracking. Resting and feeding behaviors varied with fluctuating water levels, with aquatic vegetation playing a more significant role in their diet than previously assumed. These findings provide valuable insights into hippo ecology and can help predict how they may respond to environmental changes, particularly in regions experiencing increasing human pressures.

## Teaser

This study provides the first observational 24-h activity budget of hippos and the first behavioral data from Botswana. Behavior varied by time of day, season, study area, and age class, following a clear circadian rhythm with peaks at sunrise, sunset, midday, and midnight. Contrary to the common view that hippos rest in water by day and graze all night, they fed during only a quarter of the night and were equally active during the day.

Animal activity budgets describe a series of trade-offs, where time spent on one activity limits the ability of an individual to perform another beneficial activity (Hamel and Côté 2008). Investigating these activities reveals how animals allocate time, providing insight into energy needs and habitat preferences (Prinsloo 2016; Rimbach et al. 2016; Pęksa and Ciach 2018; Fraser et al. 2019). The behavior of a species, particularly their activity budget, can vary due to a range of factors including population density, time of day, season, climate, resource availability, group composition, predation risk, inter-individual variation (e.g., personality), human disturbance, age, and sex (Timbuka 2012; Majolo et al. 2013; Owen-Smith and Goodall 2014; McQualter 2016; Mekonen and Hailemariam 2016; Rimbach et al. 2016; Pęksa and Ciach 2018; Fraser et al. 2019)—and sampling across these factors provides a more comprehensive understanding of activity budgets. Furthermore, examining how individuals respond to different environmental conditions reveals their ability to adapt to manmade or natural changes to their habitat (Owen-Smith and Goodall 2014; Rimbach et al. 2016; Pęksa and Ciach 2018).

Knowledge of Common Hippopotamus (*Hippopotamus amphibius*, hereafter referred as hippo) behavior is limited, particularly regarding their activity budgets (Eltringham 1993a) due to the difficulty of making extensive direct observations of them because they are primarily nocturnal, aquatic, dangerous, lack obvious individually identifiable features, and often inhabit areas that are difficult to access (Karstad 1984; Barklow 1997; Krueger 1997; Eltringham 1999; Lewison and Carter 2004; Maust-Mohl et al. 2015; Prinsloo 2016). To date, our understanding of diurnal hippo activity budgets is taken from 4 observational studies from the Ivory Coast, Tanzania, Ethiopia, and South Africa (Bouché 2004b; Timbuka 2012; Mekonen and Hailemariam 2016; Prinsloo 2016), with varying levels of sampling intensity and detail. In addition, the 24-h behavior of hippos in Kenya was derived from GPS tag movement data, although an activity budget was not the explicit purpose of the study (Nuñez 2017). Additional studies have explored short-term behaviors like communication and aggression (Karstad and Hudson 1986; Barklow 2004; Blowers et al. 2010; Maust-Mohl et al. 2015), with social behavior relatively well understood due to descriptive research in Uganda in the 1970s (Klingel 1991a, 1991b, 2013). Hippo behavior is also mentioned incidentally in studies on populations, ecology, physiology, and anatomy (e.g., Luck and Wright 1964; Laws and Clough 1966; Viljoen and Biggs 1998; Harrison et al. 2008).

What is lacking from most hippo activity studies is an understanding of how behavior changes over temporal and spatial scales. Many authors have collected data during short periods of the year or aggregated data across months, omitting potential seasonal variation (Bouché 2004a; Mekonen and Hailemariam 2016; Prinsloo 2016; Nuñez 2017), while others have restricted studies to a single pod (Prinsloo 2016). Previous authors have examined different focal activities—all recording resting, feeding, and moving (Timbuka 2012; Mekonen and Hailemariam 2016; Prinsloo 2016; Nuñez 2017). However, Timbuka (2012) did not record aquatic movement, despite its prevalence (Blowers et al. 2012; Mekonen and Hailemariam 2016) while Bouché (2004a) did not describe the activities he recorded and Nuñez (2017) could not differentiate between various aquatic activities. The observational studies all prioritized detailed sampling of social behaviors (e.g., yawning, vocalizations, ear-flicking), which represent only a small portion of hippo activity and did not distinguish between resting in aquatic or terrestrial habitats, leading to a superficial understanding of habitat use. Even though hippos are primarily nocturnal, most studies have not attempted to investigate nocturnal activity budgets (Timbuka 2012; Mekonen and Hailemariam 2016; Prinsloo 2016), or their attempts were unsuccessful (Bouché 2004a). By extracting data from GPS tags, without observing hippos directly, Nuñez (2017) captured nocturnal behavior, albeit using broad activity categories. To fully understand hippo behavior, food requirements, and ecology, it is essential to study their nocturnal activity because most energetically demanding behaviors occur at night (Bouché 2004a; Lewison and Carter 2004; Prinsloo 2016).

The aims of this study were to (i) quantify the 24-h activity budget of hippos; (ii) evaluate how behavior changed over the day and varied seasonally; (iii) examine how behavior varied between different areas; and (iv) between age classes. This study presents the first 24-h observational activity budget of hippos and the first behavioral data from Botswana.

## Methods

### Study area.

**Fig. 1.**
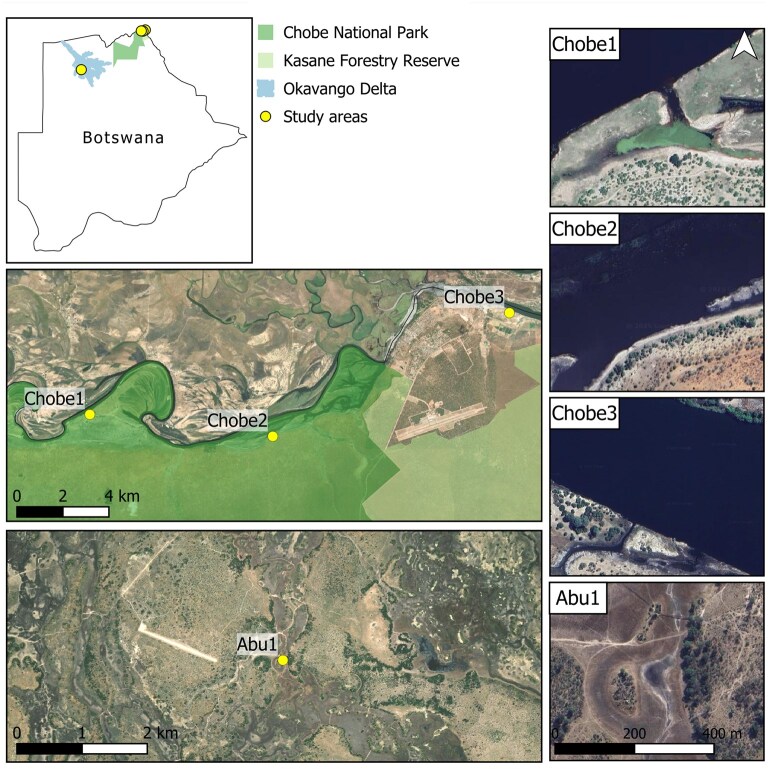
Study areas within the Abu Concession and Chobe District (Kasane township and Chobe National Park) in northern Botswana. Note: background satellite imagery is modified Copernicus Sentinel data processed by the European Space Agency.

This study was conducted within northern Botswana between 14 August 2017 and 11 October 2018 in 2 regions: the Abu Concession in the Okavango Delta (the Delta), Ngamiland District; and the Chobe River in the Chobe District (Fig. 1) (Table 1). The Delta is a Ramsar-listed Wetland of International Importance (Ramsar 2014), a World Heritage Site (UNESCO 2014), and crucial to Botswana’s tourism industry (Wolski and Murray-Hudson 2006; Milzow et al. 2009). The Abu Concession is a wildlife management area used for non-consumptive tourism (e.g., photography).

Our study area within the Abu Concession (Abu1; −19.4181°S, 22.5676°E) was located near a well-used bridge and frequently visited by tourists from nearby lodges. Although data were also collected from a second site in Abu, they were excluded from this manuscript due to limited habituation of the hippos and the availability of data from only a single season.

The Chobe riverfront extends along northern Chobe National Park and the Kasane/Chobe Forest Reserves, continuing past Kasane township. Within Chobe National Park, the riverfront is subject to intensive tourism, sometimes considered excessive (van der Sluis et al. 2017). We studied 3 areas: Chobe1 (−17.8282°S, 25.0293°E); Chobe2 (−17.8339°S, 25.1025°E) within Chobe National Park, both heavily visited by tourists; and Chobe3 (−17.7857°S, 25.1889°E) within Kasane, adjacent to undeveloped private land but near farms and a shopping complex. Initially unfenced, Chobe3 was often visited by townsfolk, but after fencing in October 2017, visits declined.

Northern Botswana has a semi-arid climate with wet summers (October–April) (McCarthy and Ellery 1993; Okavango Research Institute 2019). The Delta and Chobe River are flood pulsed systems subject to an annual flood event, predominantly fed by the Okavango (Wolski et al. 2008) and Zambezi Rivers (Pricope 2013; van der Sluis et al. 2017). Local rainfall is asynchronous with flooding, with floods peaking in July to September (Delta) and April to May (Chobe; McCarthy et al. 2000; Gumbricht et al. 2004; Tooth and McCarthy 2007; Pricope 2013). To examine seasonal and flood-related effects on hippo behavior, we collected data in 3 seasons: wet season (low flood), minimal flooding; dry season (high flood), peak flooding; and dry season (med-low flood), receding flooding (Table 1).

The onset of the wet season was defined by the first rains >10 mm and ended with the last rains of the season and varied annually. In Abu, the 2017 dry season (med-low flood) spanned October to 4 December (first rains >10 mm), followed by the wet season (low flood) until 30 March 2018 (last rains). The dry season (high flood) ran from July to September 2018, followed by the dry season (med-low flood), which ceased on 21 October 2018 due to the arrival of rains. In Chobe, the 2017 dry season (med-low flood) ran from August to 20 October (first rains >10 mm), followed by the wet season (low flood) until 5 April 2018 (last rains). The dry season (high flood) lasted until late July 2018, followed by the dry season (med-low flood).

### Sampling technique and schedule.

Observations were conducted in full day or full night sessions wherever possible. Diurnal sessions began at or just after sunrise and ended before sunset, while nocturnal observations began before sunset and ended after sunrise. Observations commenced 10 min after arrival in order to minimize any disturbance effects of the vehicle. Observations were conducted from a vehicle in a vantage point chosen to provide an optimal field of vision but at a distance to reduce disturbing hippos. Bushnell Excursion binoculars (8 × 42, FOV 426 ft) were used for distant observations, and nocturnal observations were aided by a handheld, red-filtered spotlight, and a Night Owl Explorer Pro night scope.

Behavioral data were recorded using instantaneous scan sampling (Altmann 1974) every 5 min, with the activity of all visible hippos recorded. Not all hippos were visible in every scan due to submersion or movement out of the area. Hippos were classified into 1 of 9 mutually exclusive activities (Table 2; Supplementary Data SD1). The age class (juvenile, subadult, or adult) of each individual hippo was recorded if possible (otherwise the hippo was recorded as unknown). Hippos judged as less than 1/2 the length of the largest hippo (typically the dominant male; Laws 1968; Skinner et al. 1975) were classed as juveniles; subadults were between 1/2 and 2/3 the length of the largest hippo; and adults were over 2/3 the length of the largest hippo. Distinguishing adjacent hippo pods was difficult, especially in the Chobe River, where the continuous habitat facilitated frequent group changes. In contrast, hippos in the Okavango Delta occupied discrete lagoons, making pod identification more straightforward. To ensure comprehensive data collection, all observable hippos were recorded regardless of spatial separation. For each area and season, we collected data during 3 diurnal and 3 nocturnal sessions. This sampling effort was considered appropriate, yielding approximately 430 observations per individual per area, season, and time of day—more than the 255 observations found sufficient to estimate common behaviors in another ungulate study (Wilson et al. 2008). Furthermore, our effort aligns with other hippo studies: Timbuka (2012) used 5-min scans for 3 h/day over 2 d per month; Mekonen and Hailemariam (2016) observed a pair of hippos for 5 d/month; Prinsloo (2016) sampled for 13 d but only every 30 min. Observations were not conducted on consecutive days unless required due to incomplete sessions (this mostly occurred during nocturnal observations).

**Table 1.** Data collection dates, average daily temperatures (range in brackets), and total rainfall for each region for each season.

Season	Region	Dates	Average max temp (°C)	Average min temp (°C)	Rainfall (mm)
Dry season (med-low flood)	Chobe	Aug–Oct 2017 Sept 2018	33.2 (28.6–36.6)	13.5 (6.2–21.2)	0.0
	Abu	Oct 2018	35.2 (34.4–36.0)	11.9 (8.9–16.0)	0.0
Wet season (low flood)	Chobe	Jan 2018	33.1 (28.4–36.4)	19.3 (17.2–21.3)	73.5
	Abu	Feb–Mar 2018	29.8 (23.0–33.5)	18.9 (16.6–20.9)	54.9
Dry season (high flood)	Chobe	Apr–May 2018	29.0 (27.4–30.5)	11.9 (7.6–17.9)	0.0
	Abu	Jul–Aug 2018	31.8 (28.2–36.1)	7.4 (3.2–11.8)	0.0

Weather variables correspond to exact dates of data collection and for Abu were from Shakawe Station (−18.37°S, 21.83°E, approximately 140 km from study area) and for Chobe from Kasane Station (−17.82°S, 25.15°E, approximately 4 to 13 km from study area; Government of Botswana 2019).

**Table 2.** Description and codes of activities assigned to all visible hippos.

Activity and code	Description
Resting deep aquatic (RDA)	Not moving, more than 2/3 of body submerged in water
Resting shallow aquatic (RSA)	Not moving, less than 1/3 of body submerged in water
Resting terrestrial (RT)	Not moving on land
Moving aquatic (MA)	Walking, porpoising, diving, or surfacing in the water without any other target activity (e.g., feeding)
Moving terrestrial (MT)	Moving on land without any other target activity (e.g., feeding)
Feeding aquatic (FA)	Consuming vegetation growing in water
Feeding terrestrial (FT)	Consuming vegetation growing on land
Social (S)	Any social behavior included yawning, mating, fighting, playing, tail paddling, dung paddling. Vocalizations were noted but not recorded as an activity as it was difficult to distinguish which hippos were vocalizing
Other (X)	Unknown behavior or one that did not fit into other categories. A brief description was recorded

During diurnal sessions, all hippos rarely moved out of sight, but if they did the vehicle was repositioned. After sunset, hippos tended to move away in the water or leave the water singly or in small groups. Observations continued until the last hippo left the area. Hippos were then followed by vehicle keeping as many individuals in sight as possible. It was common to lose sight of hippos at night; in such cases, the observer remained in place for at least 2 scans (10 min) to determine whether the hippos were submerged or behind vegetation. If hippos were still suspected nearby (e.g., based on vocalizations or movement), the observer continued waiting until no further signs were detected. If necessary, the observer then searched systematically beginning at the last known location and expanding outward until the hippos were relocated. Upon finding a hippo, scans resumed after a 10-min delay to minimize disturbance. This process was frequently required during nocturnal observations.

### Analysis

Each scan was classified as diurnal or nocturnal based on the time relative to sunrise/sunset (sunriset function from “maptools” package; Bivand and Lewin-Koh 2019). Due to difficulties in age classification, hippos were grouped into a single category for most of the analyses. Age-based differences in behavior were only considered by examining plotted data. For each scan, the total number of observed hippos was calculated, and the proportion of hippos doing each activity was calculated, where all activity proportions for a scan summed to 1.

We tested the effect of area, season, time of day, and the interaction between season and time of day on hippo behavior by fitting a series of generalized additive models (binomial distribution), separately for each activity, using the gam function from the “mgcv” package (Wood 2017). Scan proportion was the response variable, and the total count of hippos for the scan was included as a weight in each of the models. For time of day, we fit a cyclic cubic regression spline to account for the circularity of the variable. Model selection was conducted using the double penalty approach, which allows the model to penalize and shrink smooth terms, including shrinking them toward 0 (effectively removing non-informative terms from the model; Wood 2017). This method results in parsimonious models without the need for stepwise selection and reduces the risk of overfitting. Significance of explanatory variables was assessed using the summary function, and model fit was evaluated with “gam.check.” Predicted probabilities of hippo activities were generated using the predict function. All statistics were conducted using the R computing environment (version 3.5.2; R Core Team 2018).

## Results

We made a total of 8,181 scans (5,558 diurnal; 2,623 nocturnal), resulting in 17,376 activity records (Supplementary Data SD2 and SD3). Seasonally, for each area, we averaged 505 diurnal scans (range 381 to 896) and 262 nocturnal scans (range 135 to 400). The discrepancy in diurnal and nocturnal scan numbers was primarily due to the difficulty of following hippos at night, as well as the earlier-than-expected onset of the wet season in Abu that prevented the collection of nocturnal data for the dry season (med-low flood). Chobe3 could not be sampled during the dry season (high flood). The dry season (med-low flood) was sampled twice (2017 and 2018) for the 3 Chobe areas. The mean number of hippos observed per scan was 13 during the day (range = 1 to 110) and 5 at night (range = 1 to 41), with Chobe2 having the most hippos and Abu1 the fewest (Supplementary Data SD4). Nocturnal observations included fewer individuals because of the reduced visibility at night, the tendency of hippos to disperse while grazing, and that hippos were sometimes disturbed by the vehicle while on land.

### Daily activity budget.

**Fig. 2.**
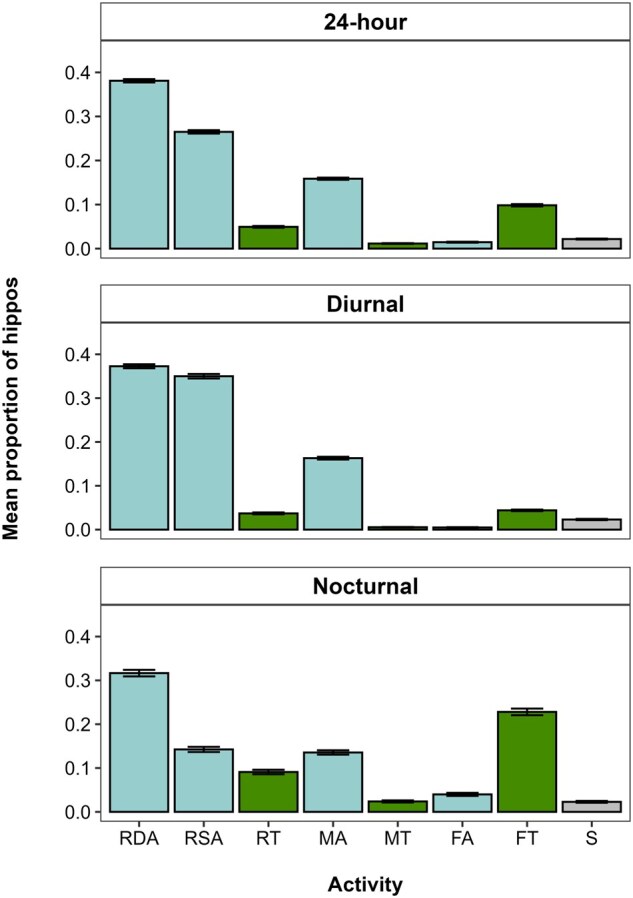
Twenty-four-hour, diurnal, and nocturnal activity budgets of hippos combined from all seasons and areas. Colors indicate location (blue, aquatic; green, terrestrial; gray, unspecified). Error bars represent standard error.

Combining all data, the average 24-h activity budget of hippos (Fig. 2) was 69.2% resting—35.5% resting deep aquatic (RDA), 28.3% resting shallow aquatic (RSA), 5.4% resting terrestrial (RT); 16.5% moving—15.4% moving aquatic (MA), 1.1% moving terrestrial (MT); 11.9% feeding—10.3% feeding terrestrial (FT), 1.6% feeding aquatic (FA); and 2.3% social activity. Examining diurnal/nocturnal observations separately (Fig. 2), hippos rested during the majority of the day (76.0%: 37.3% RDA; 35.0% RSA; 3.7% RT), with 16.8% moving (16.3% MA; 0.5% MT), 4.9% feeding (4.4% FT; 0.5% FA), and 2.3% social activity. The majority (89.1%) of these activities occurred in the water, with 8.6% occurring on land (social activity not included). Resting was also the leading activity (55.0%) at night (31.7% RDA, 14.2% RSA, 9.1% RT), but feeding was the second most common activity (26.8%: 22.8% FT; 4.0% FA), followed by moving (15.9%: 13.5% MA; 2.4% MT) and 2.3% social activity. Even at night, hippos were mostly in the water (63.4%), with 34.3% of the time on land. The activity budgets for each area, for each season, divided by day and night are provided in Supplementary Data SD5, as are notable field observations (Supplementary Data SD6).

### Effect of time of day and season on hippo behavior.

**Fig. 3.**
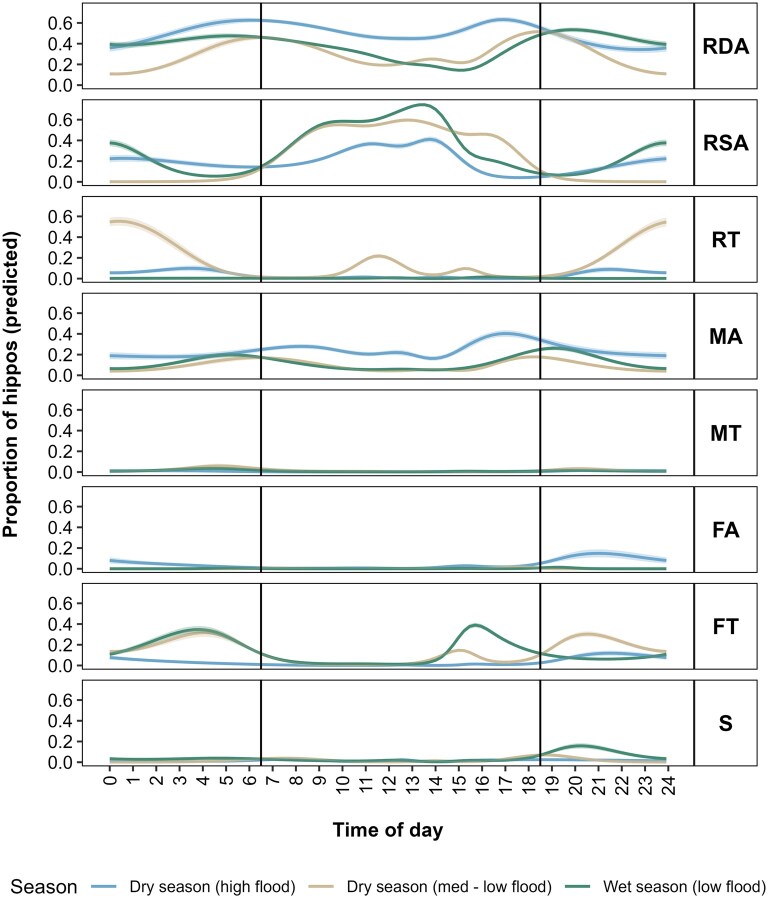
Proportion of hippos exhibiting each behavior in each season throughout the day, predicted from GAM models. Vertical black lines denote approximate sunrise and sunset times.

The interaction between time of day and season significantly affected the proportion of hippos engaged in each behavior (Supplementary Data SD7 to SD14). Time of day significantly influenced all activities in all seasons (*P *< 0.05), except for FA during the wet season (low flood), where the effect was marginal (*P *= 0.060). These results indicate that hippos exhibit a well-defined circadian rhythm, which varies seasonally (Fig. 3). RDA peaked around sunrise and sunset in all seasons and was most common in the dry season (high flood), especially during the day. RSA followed an opposite pattern, peaking midday and dipping at sunrise and sunset with another smaller peak around midnight in the wet season (low flood) and, to a lesser extent, in the dry season (high flood). RT was generally rare, except in the dry season (med-low flood) where it peaked around midnight, surpassing RSA and RDA, with additional peaks during the middle of the day. MA peaked around sunrise and sunset and was most common during the dry season (high flood). MT was infrequent, peaking slightly before sunrise and after sunset in the dry season (med-low flood) and wet season (low flood). FT peaked before sunrise and again in the late afternoon during the low-flood seasons, with an extra post-sunset peak in the dry season (med-low flood). FT was lowest during the dry season (high flood) with only a small peak after sunset. FA was rare overall, but most frequent in the dry season (high flood), peaking after sunset. Social activity remained low across all periods, with a peak around and after sunset and was most common in the wet season (low flood) and least in the dry season (high flood).

### Variation in behavior among study areas.

**Fig. 4.**
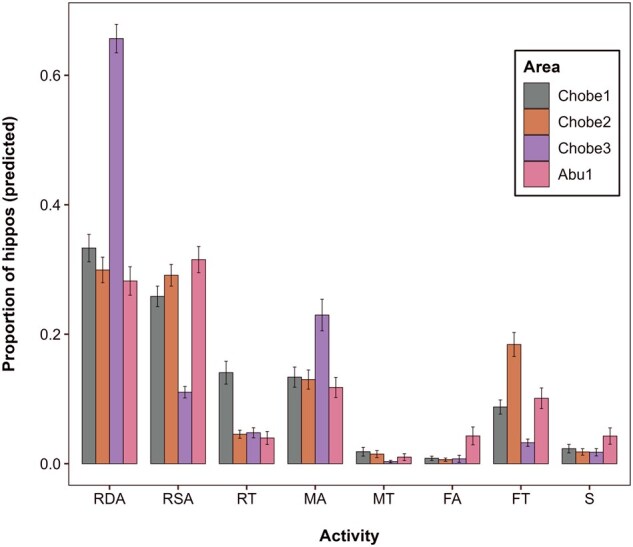
Proportion of hippos exhibiting each behavior in each area, predicted from GAM models. Error bars represent 95% confidence intervals.

There were some substantial differences in hippo behavior between the study areas (Fig. 4). Hippos in Chobe3 spent more time RDA and MA and less time RSA and FT than in the other areas. Chobe1 showed a notable amount of RT, which was much lower in other areas. Hippos in Abu1 had the highest proportion of FA. Social behavior was generally low across all areas but was higher in Abu1 than in other locations.

### Variation in behavior among age classes.

**Fig. 5.**
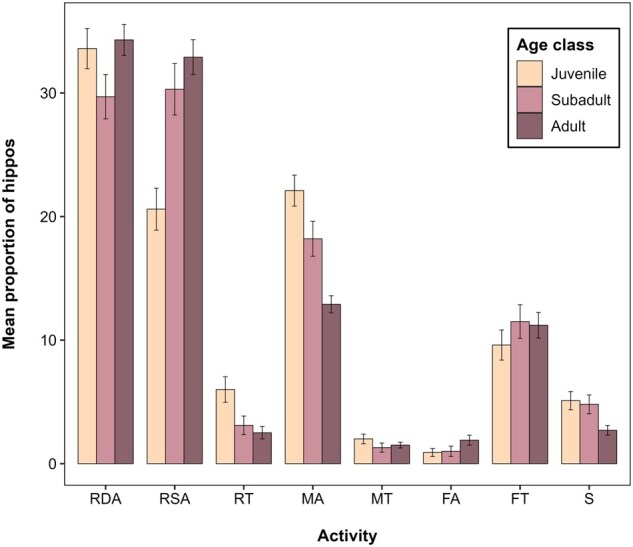
Proportion of hippos exhibiting each behavior based on age. Error bars represent standard error.

Hippo behavior varied by age class (Fig. 5). Juvenile hippos rested least (60.7%), followed by subadults (63.3%) and then adults (69.7%). Juveniles, subadults, and adults spent similar amounts of time RDA, but juveniles RSA less than subadults and adults, and RT more. Juveniles fed least (9.7%) compared to subadults (11.5%) and adults (11.2%), and juveniles and subadults FA much less than adults. Juveniles and subadults engaged in similar levels of social activity (4.8% and 5.0%, respectively), both higher than adults (2.7%).

## Discussion

Our results challenge the long-standing dominant narrative that hippos exclusively rest in water by day and graze on land all night (Clough 1967; Field 1970; Hoven 1978; Owen-Smith 1988; Eltringham 1993b, 1999; Barklow 1997; Klingel 2013). Although other recent studies (Timbuka 2012; Mekonen and Hailemariam 2016; Prinsloo 2016) also documented high levels of non-resting daytime activity (Table 3), they did not frame their findings as notable behavioral deviations. This, combined with a lack of nocturnal observations, has allowed the simplistic narrative of “diurnal aquatic resting/nocturnal terrestrial feeding” to persist. Overall, hippos in this study spent around a quarter of the day active and at night spent only a quarter of their time feeding, often resting instead. Notably, in some areas and seasons, hippos spent almost: 20% of the day grazing on land, Chobe2 in the wet season (low flood); 16% of the day basking on land in the sun, Chobe1 in the dry season (med-low flood); and 47% of the night resting on land, Chobe2 in the dry season (med-low flood).

**Table 3.** Comparison of diurnal and 24-h activity budget of hippos from this study with 4 other studies. Activity codes were adapted for consistency.

Time	Source	Country	Activity			
			Resting	Moving	Feeding	Social
Diurnal	This study	Botswana	75.9%	17.3%	4.6%	2.2%
	Mekonen (2016)	Ethiopia	42.5%	34.2%	19.6%	3.7%
	Timbuka (2012)	Tanzania	53.3%	18.1%	19.3%	8.9%
	Prinsloo (2016)	South Africa	79.4%	10.4%	2.2%	7.9%
			**Aquatic**	**Resting land**	**Feeding land**	**Moving land**
24 h	This study	Botswana	81.9%	4.9%	9.8%	1.1%
	Nuñez (2017)	Kenya	60.7%	9.0%	20.6%	9.7%

Our results are most aligned with those from activity budget studies in South Africa (Prinsloo 2016), which shares a similar climate to Botswana. Over a 24-h period, hippos in Kenya (Nuñez 2017) spent twice as much time on land compared to our study, possibly due to environmental differences or reduced observer disturbance (behavior was derived from GPS tag movement data). Although the diurnal activity levels we observed fall within the range reported in other hippo activity budgets (Table 3), many of these studies did not distinguish between behaviors occurring on land or in water, limiting the potential for direct comparisons.

In this study, hippos regularly engaged in extended terrestrial resting and feeding during the day, even in exposed unshaded locations under high ambient temperatures (up to 35 °C; Supplementary Data SD6). This result contradicts widely held assumptions that hippos rely on water during the day to prevent skin desiccation and due to their high evaporative water loss (EWL)—an idea largely rooted in the early work of Luck and Wright (1964). Those results have been erroneously interpreted as hippos exhibiting the highest EWL of any mammal (Jablonski 2004; Timbuka 2012), leading to the conclusion that a hippo on land in hot weather risks rapid dehydration and that its skin will crack or desiccate if exposed to air (Field 1970; Estes 1991; Eltringham 1993b, 1999; Jablonski 2004; Blowers 2008; Mazza 2015; Mpemba 2015; Prinsloo 2016; Stommel et al. 2016; Lewison and Pluháček 2017), although Luck and Wright (1964) did not report desiccation or cracking. We observed no signs of skin cracking or distress from heat exposure during terrestrial basking, suggesting that water use during the day may not be exclusively driven by physiological constraints but also by behavioral responses to perceived threats. Hippos often rapidly return to deep water when disturbed (Supplementary Data SD6; Clarke 1953; Pooley 1967; Klingel 1991a; Eltringham 1999; Onyeanusi 2006) and were more likely to engage in terrestrial behaviors, where they had access to human-inaccessible areas.

Crucially, this study contributes new insights into nocturnal behavior, a period that has never been directly observed and described—although note that Nuñez (2017) derived nocturnal behavior from GPS tag movement data. Our results challenge the long-standing assumptions that hippos graze from sunset to sunrise as is commonly reported (Luck and Wright 1964; Field 1970; Hoven 1978; Eltringham 1993b; Barklow 1997; Eltringham 1999; Klingel 2013), but instead follow a pattern of feeding before sunrise and after sunset, while much of the middle of the night was spent resting either in water or on land. Although hippos were sometimes disturbed on land, potentially discouraging grazing, individuals that appeared undisturbed still fed for approximately average durations. This pattern suggests that disturbance alone does not fully explain the low feeding rates observed. Hippos have lower energetic demands than similar-sized mammals (Eltringham 1993b, 1999; Lewison and Carter 2004; Klingel 2013), which could further explain the relatively short feeding times.

Season strongly influenced behavior as shown also in other studies (Timbuka 2012; Mekonen and Hailemariam 2016), although Botswana’s unique seasons (where flooding and rainfall are asynchronous) complicate direct comparisons. During the dry season (high flood), hippos rested mostly in deep water, likely due to fewer shallow water resting areas. We also often recorded movement in water, potentially incorrectly classifying hippos resting in deep water and raising their head to breathe as MA instead of resting. In contrast, in the dry season (med-low flood) hippos often rested on land, possibly because receding floodwaters provided safe and accessible sandbanks. Resting on land was limited during the wet season (low flood) despite lower water levels, possibly due to biting insects (we observed numerous hippos with skin covered with bumps; Eltringham 1999) or rainfall (Klingel 2013). Overall resting time remained relatively constant across seasons, suggesting that hippos adjust the location rather than duration of rest in response to seasonal factors.

Feeding on land was lowest during the dry season (high flood); hippos may have been eating less because the easily accessible grass on the floodplains was submerged or alternatively may have been grazing in woodlands where they were harder to observe. Timbuka (2012) also reported reduced feeding in the dry season, though others suggest that hippos feed longer at night, and continued to feed in the day in dry conditions (Mugangu and Hunter 1992; Klingel 2013). When food is scarce, low in quality, or far away, animals must resolve spending more energy attempting to meet intake requirements, or prioritizing conservation of energy by minimizing time spent foraging (Scotcher et al. 1978; Dasilva 1992; Timbuka 2012). In contrast, feeding on aquatic vegetation peaked due to increased growth during this season. The amount of time spent feeding was similar in the 2 lower flood seasons, likely due to rain-promoted grass growth in the wet season (low flood; Timbuka 2012) and growth where the flood had receded in the dry season (med-low flood; Bonyongo 2009). During these seasons, hippos consumed more terrestrial grasses than aquatic vegetation as the relative availability of these food sources changed.

Activity budgets varied among sites, likely reflecting variations in available habitat. For example, Chobe1 and Chobe2, situated along the same river system only 8 km apart had some strong differences in behavior. Hippos from Chobe1 rested on land more than Chobe2 and the other areas, likely due to the presence of a large sandbank adjacent to the lagoon inaccessible to vehicles, where hippos frequently basked during the day (Supplementary Data SD6). This preference for sandy resting sites aligns with previous studies (Ansell 1965; Klingel 1991b; Barklow 1997; Onyeanusi 2006). In contrast, during the day, hippos in Chobe2 rarely rested on land because the only sandy area was directly adjacent to a busy vehicle track. However, at night, it was common for hippos to rest on this patch of sand when there was no vehicle traffic. Chobe2 had the highest feeding activity, particularly diurnally in the wet season (low flood), where there was a vast area of exposed floodplain that was inaccessible to vehicles (Timbuka 2012; Prinsloo 2016). Hippos in Chobe3 did not rest in shallow water as often as the other areas and limited diurnally terrestrial feeding likely due to their location in a deeper, faster-flowing section of the Chobe River, reducing opportunities for shallow resting and with no exposed floodplain to feed in. Our results suggest that even habituated hippos likely still alter their behaviors due to human presence. The activity budgets documented in this study are likely reflective of broader behavioral patterns among hippos in Botswana. These findings may also be cautiously extrapolated to populations in other countries with similar climates and low levels of human persecution, provided that small-scale habitat differences and local disturbance are taken into account.

Aquatic feeding, although generally uncommon, was observed across all sites and could account for a substantial portion of nocturnal activity up to 27% at Abu1 during the dry season (high flood; more information on FA is provided in Supplementary Data SD6). There are previous reports that hippos may opportunistically consume aquatic vegetation (Taylor 1976; Hoven 1978; Mugangu and Hunter 1992; Harrison et al. 2008; Klingel 2013; Mekonen and Hailemariam 2016; Prinsloo 2016), yet our results suggest that aquatic vegetation can be a significant food source and contradict the claim that the hippo “does not eat aquatic vegetation to any extent” (Eltringham 1993b:47, 1999:3). The higher rates of aquatic feeding in Abu likely reflect both local habitat conditions (e.g., shallow, slow-moving water encouraging aquatic vegetation growth) and limited availability of terrestrial forage due to seasonal inundation.

Social behavior of hippos is often underestimated because much occurs underwater (Barklow 1997, 2004; Prinsloo 2016). The generally low levels of social activity observed in this study may be due to abundant water in Botswana (particularly in the dry season—high flood) and low hippo densities, reducing male competition and aggression (Olivier and Laurie 1974; Karstad and Hudson 1986; Bouché 2004a; Blowers et al. 2010; Timbuka 2012; Klingel 2013).

Behavioral differences by age were evident but subtle. Juveniles rested less in shallow water likely due to their inability to stand in deeper areas (Klingel 2013), and more on land, possibly to reduce heat loss in cold water (Taylor in Klingel 2013). They also fed less than adults, often resting close to feeding mothers rather than grazing (Bruton 1978; Timbuka 2012; Klingel 2013). Subadults and adults fed at similar rates, both more than juveniles, who likely still suckle (categorized as “other”; Supplementary Data SD6), although even young juveniles grazed (Clough 1967; Laws and Clough 1966; Young 1966). Social behavior was more frequent among juveniles and subadults, including play-fighting and chasing (Bouché 2004a; Timbuka 2012). Further categorization of social behavior into aggressive and non-aggressive codes might have revealed stronger differences from adults, particularly males (Karstad 1984; Karstad and Hudson 1986).

This study contributes significant new knowledge about this charismatic megafauna species by directly documenting nocturnal activity and by highlighting the underappreciated role of terrestrial behavior during the day. These findings support a more comprehensive and nuanced understanding of hippo behavior and habitat use. Variations in activity budgets across seasons and sites indicate behavioral adaptability but also highlight their sensitivity to human presence. Hippos feed and rest on land during the day when sandy and grassed areas are available and free from human disturbance, underscoring the importance of regulating vehicle access near hippo pods. In addition, protecting aquatic habitats so that they maintain a mix of shallow and deep water is vital to support resting, movement, and social behaviors.

## Supplementary Material

gyaf068_Supplementary_Data

## Data Availability

Data and code are available from the corresponding author on reasonable request.

## References

[gyaf068-B1] Altmann J. 1974. Observational study of behavior: sampling. Behaviour 49(3):227–267. 10.1163/156853974X00534 4597405

[gyaf068-B3] Ansell WFH. 1965. Hippo census on the Luangwa River. The Puku 32(3):647–655.

[gyaf068-B5] Barklow WE. 2004. Amphibious communication with sound in hippos, *Hippopotamus amphibius*. Animal Behaviour 68(5):1125–1132. 10.1016/j.anbehav.2003.10.034

[gyaf068-B6] Barklow WE. 1997. Some underwater sounds of the hippopotamus (*Hippopotamus amphibius*). Marine and Freshwater Behaviour and Physiology 29(1-4):237–249. 10.1080/10236249709379008

[gyaf068-B9] Bivand R , Lewin-Koh N. 2019. maptools: tools for handling spatial objects. R package version 0.9-5. https://cran.r-project.org/package=maptools.

[gyaf068-B10] Blowers TE. 2008. Social grouping behaviors of captive female *Hippopotamus amphibius* [master’s thesis]. [Florida (USA)]: University of Central Florida.

[gyaf068-B11] Blowers TE , Waterman JM, Kuhar CW, Bettinger TL. 2010. Social behaviors within a group of captive female *Hippopotamus amphibius*. Journal of Ethology 28(2):287–294. 10.1007/s10164-009-0184-6

[gyaf068-B12] Blowers TE , Waterman JM, Kuhar CW, Bettinger TL. 2012. Female Nile hippopotamus (*Hippopotamus amphibius*) space use in a naturalistic exhibit. Zoo Biology 31(2):129–136. 10.1002/zoo.20366 22535694

[gyaf068-B13] Bonyongo MC. 2009. Okavango river basin technical diagnostic analysis: environmental flow module. Botswana: OKACOM.

[gyaf068-B14] Bouché PJC. 2004a. Ecology and activity of hippopotamuses in the White Bandama River, Ivory Coast. [accessed 8 May 2017]. https://www.researchgate.net/publication/311921686_Ecology_and_activity_of_Hippopotamus_in_the_White-Bandama_River_Ivory_Coast

[gyaf068-B15] Bouché PJC. 2004b. Hippopotamus of the W-Arli-Pendjari-Oti-Mandouri-Keran ecosystem. West Africa. Status, distribution and conservation issues. Hippo Pigs Peccaries News 4(1):14–19.

[gyaf068-B16] Bruton MN. 1978. Recent mammal records from eastern Tongaland in Kwazulu, with notes on hippopotamus in Lake Sibaya. Lammergeyer 24:19–27.

[gyaf068-B18] Clarke JR. 1953. The hippopotamus in Gambia, West Africa. Journal of Mammalogy 34(3):299–315. 10.2307/1375838

[gyaf068-B19] Clough G. 1967. Reproduction in the hippopotamus *Hippopotamus amphibius* (Linn.) [dissertation]. [Cambridge (UK)]: University of Cambridge.

[gyaf068-B20] Dasilva GL. 1992. The Western Black-And-White Colobus as a low energy strategist: activity budgets, energy expenditure and energy intake. Journal of Animal Ecology 61(1):79–91. 10.2307/5511

[gyaf068-B21] Eltringham SK. 1993a. The afrotropical hippopotamuses (Hippopotamus and Hexaprotodon): review of priorities for conservation action and future research on hippopotamuses. In: Oliver WL, editor. Pigs, peccaries and hippos: status survey and conservation action plan. Gland (Switzerland): IUCN; p. 61–65.

[gyaf068-B22] Eltringham SK. 1993b. The afrotropical hippopotamuses (Hippopotamus and Hexaprotodon): the common hippopotamus (*Hippopotamus amphibius*). In: Oliver WL, editor. Pigs, peccaries, and hippos: status survey and conservation action plan. Gland (Switzerland): IUCN; p. 43–55.

[gyaf068-B23] Eltringham SK. 1999. The hippos: natural history and conservation. London (UK): Academic Press.

[gyaf068-B24] Estes RD. 1991. The behavior guide to African mammals. Berkeley (CA, USA): University of California Press.

[gyaf068-B25] Field CR. 1970. A study of the feeding habits of the hippopotamus (*Hippopotamus amphibius* Linn.) in the Queen Elizabeth National Park, Uganda, with some management implications. Zoologica Africana 5(1):71–86. https://www.tandfonline.com/doi/abs/10.1080/00445096.1970.11447382

[gyaf068-B26] Fraser ZL , Culloch RM, Twiss SD. 2019. As clear as day: nocturnal activity differs from diurnal activity in a temporally constrained capital breeder. Behaviour 156(10):997–1016. 10.1163/1568539X-00003553

[gyaf068-B27] Government of Botswana. 2019. Daily weather data. Kasane and Shakawe Stations: Bureau of Meteorology.

[gyaf068-B28] Gumbricht T , Wolski P, Frost P, McCarthy TS. 2004. Forecasting the spatial extent of the annual flood in the Okavango Delta, Botswana. Journal of Hydrology 290(3-4):178–191. 10.1016/j.jhydrol.2003.11.010

[gyaf068-B29] Hamel S , Côté SD. 2008. Trade-offs in activity budget in an alpine ungulate: contrasting lactating and nonlactating females. Animal Behaviour 75(1):217–227. 10.1016/j.anbehav.2007.04.028

[gyaf068-B30] Harrison ME , Kalindekafe MP, Banda B. 2008. The ecology of the hippopotamus in Liwonde National Park, Malawi: implications for management. African Journal of Ecology 46(4):507–514. 10.1111/j.1365-2028.2007.00887.x

[gyaf068-B32] Hoven WVAN. 1978. Digestion physiology in the stomach complex and hindgut of the hippopotamus (*Hippopotamus amphibius*). South African Journal of Wildlife Research 8:59–64.

[gyaf068-B33] Jablonski NG. 2004. The hippo’s tale: how the anatomy and physiology of Late Neogene Hexaprotodon shed light on Late Neogene environmental change. Quaternary International 117(1):119–123. 10.1016/S1040-618200121-6

[gyaf068-B34] Karstad EL. 1984. The ecology of hippopotami (*Hippopotamus amphibius*) in southwestern Kenya [master’s thesis]. [Alberta (Canada)]: The University of Alberta. 10.7939/r3-8nm8-cs71

[gyaf068-B35] Karstad EL , Hudson RJ. 1986. Social organization and communication of riverine hippopotami in southwestern Kenya. Mammalia 50(2):153–164. 10.1515/mamm.1986.50.2.153

[gyaf068-B37] Klingel H. 2013. *Hippopotamus amphibius* common hippopotamus. In: Kingdon J, Hoffman M, editors. Mammals of Africa: Volume VI: pigs, hippopotamuses, chevrotain, giraffes, deer and bovids. London (UK): Bloomsbury Publishing; p. 68–77.

[gyaf068-B38] Klingel H. 1991a. Sizing up a heavyweight. International Wildlife 21(5):4.

[gyaf068-B39] Klingel H. 1991b. The social organisation and behaviour of *Hippopotamus amphibius*. African Wildlife: Research and Management 73–75.

[gyaf068-B40] Krueger S. 1997. Hippopotamus underwater behavior and communication. Animal Keepers Forum 24(3):108–110.

[gyaf068-B41] Laws RM , Clough G. 1966. Observations on reproduction in the hippopotamus *Hippopotamus* *amphibius* Linn. In: Rowlands IW, editor. Comparative biology of reproduction in mammals. London (UK): Academic Press; p. 117–140.

[gyaf068-B42] Laws RM. 1968. Dentition and ageing of the hippopotamus. African Journal of Ecology 6(1):19–52. 10.1111/j.1365-2028.1968.tb00899.x

[gyaf068-B44] Lewison RL , Carter J. 2004. Exploring behavior of an unusual megaherbivore: a spatially explicit foraging model of the hippopotamus. Ecological Modelling 171(1-2):127–138. 10.1016/S0304-3800(03)00305-3

[gyaf068-B45] Lewison RL , Pluháček J. 2017. Hippopotamus Amphibius. the IUCN red list of threatened species 2017. Copenhagen (Denmark): Global Biodiversity Information Facility.

[gyaf068-B46] Luck CP , Wright PG. 1964. Aspects of the anatomy and physiology of the skin of the hippopotamus (*H. amphibius*). Quarterly Journal of Experimental Physiology and Cognate Medical Sciences 49(1):1–14. 10.1113/expphysiol.1964.sp001695 14115273

[gyaf068-B47] Majolo B , McFarland R, Young C, Qarro M. 2013. The effect of climatic factors on the activity budgets of Barbary Macaques (*Macaca sylvanus*). International Journal of Primatology 34(3):500–514. 10.1007/s10764-013-9678-8

[gyaf068-B48] Martin RB. 2005. Transboundary species project, background study, hippopotamus. Proceedings of the Transboundary Mammal Project of the Ministry of Environment and Tourism, Namibia facilitated by The Namibia Nature Foundation. Windhoek (Namibia).

[gyaf068-B49] Maust-Mohl M , Soltis J, Reiss D. 2015. Acoustic and behavioral repertoires of the hippopotamus (*Hippopotamus amphibius*). Journal of the Acoustical Society of America 138(2):545–554. 10.1121/1.4923363. 26328671

[gyaf068-B50] Mazza PPA. 2015. To swim or not to swim, that is the question: a reply to van der Geer et al. Lethaia 48(3):289–290. 10.1111/let.12129

[gyaf068-B51] McCarthy TS , Cooper GRJ, Tyson PD, Ellery WN. 2000. Seasonal flooding in the Okavango Delta, Botswana—recent history and future prospects. South African Journal of Science 96(1):25–33. 10520/AJA00382353_7717

[gyaf068-B52] McCarthy TS , Ellery WN. 1993. The Okavango Delta. Geobulletin 36(2):5–8.

[gyaf068-B54] McQualter KN. 2016. The ecology and behaviour of giraffe in northern Botswana [dissertation] [NSW (Australia)]: University of New South Wales.

[gyaf068-B55] Mekonen S , Hailemariam B. 2016. Ecological behaviour of common hippopotamus (*Hippopotamus amphibius*, LINNAEUS, 1758) in Boye wetland, Jimma, Ethiopia. American Journal of Scientific and Industrial Research 7(2):41–49.

[gyaf068-B56] Milzow C , Kgotlhang L, Bauer-Gottwein P, Meier P, Kinzelbach W. 2009. Regional review: the hydrology of the Okavango Delta, Botswana—processes, data and modelling. Hydrogeology Journal 17(6):1297–1328. 10.1007/s10040-009-0436-0

[gyaf068-B57] Mpemba J. 2015. Studies on the effects of *Hippopotamus amphibius* vectored subsidies on the ecology of aquatic ecosystem [master’s thesis]. [Morogoro (Tanzania)]: Sokoine University of Agriculture.

[gyaf068-B58] Mugangu TE , Hunter MLJ. 1992. Aquatic foraging by Hippopotamus in Zaïre: response to a food shortage? Mammalia 56(3):345–349. 10.1515/mamm.1992.56.3.345

[gyaf068-B60] Nuñez TA. 2017. Animal movement in a changing world [dissertation]. [California (USA)]: University of California.

[gyaf068-B61] Okavango Research Institute 2019. Okavango Delta: monitoring and forecasting. Maun (Botswana). http://168.167.30.198/ori/

[gyaf068-B62] Olivier RCD , Laurie WA. 1974. Habitat utilization by hippopotamus in the Mara River. African Journal of Ecology 12(4):249–271. 10.1111/j.1365-2028.1974.tb01036.x

[gyaf068-B63] Onyeanusi AE. 2006. Some behavioural characteristics of common hippopotamus (*H. amphibius* Linn. 1758) in Nigeria’s Kainji Lake National Park. International Journal of Agriculture and Rural Development 5(1):27–35. 10.4314/ijard.v5i1.2560

[gyaf068-B64] Owen-Smith N , Goodall V. 2014. Coping with savanna seasonality: comparative daily activity patterns of African ungulates as revealed by GPS telemetry. Journal of Zoology 293(3):181–191. 10.1111/jzo.12132

[gyaf068-B65] Owen-Smith RN. 1988. Megaherbivores: the influence of very large body size on ecology. Cambridge (UK): Cambridge University Press.

[gyaf068-B66] Pęksa Ł , Ciach M. 2018. Daytime activity budget of an alpine ungulate (*Tatra chamois Rupicapra rupicapra tatrica*): influence of herd size, sex, weather and human disturbance. Mammal Research 63(4):443–453. 10.1007/s13364-018-0376-y

[gyaf068-B67] Pooley AC. 1967. Bird/crocodile and bird/hippopotamus commensalism in Zululand. Ostrich 38(1):11–12. 10.1080/00306525.1967.9639469

[gyaf068-B68] Pricope NG. 2013. Variable-source flood pulsing in a semi-arid transboundary watershed: the Chobe River, Botswana and Namibia. Environmental Monitoring and Assessment 185(2):1883–1906. 10.1007/s10661-012-2675-0 22572801

[gyaf068-B69] Prinsloo AS. 2016. Aspects of the spatial and behavioural ecology of *Hippopotamus amphibius* in the Saint Lucia Estuary, KwaZulu-Natal, South Africa [master’s thesis]. [Cape Town (South Africa)]: University of Cape Town. http://doi.org/11427/20433

[gyaf068-B70] R Core Team. 2018. R: a language and environment for statistical computing. [Computer software]. Vienna (Austria): R Foundation for Statistical Computing. www.R-project.org.

[gyaf068-B71] Ramsar. 2014. Ramsar sites information service. [accessed 9 September 2019]. https://rsis.ramsar.org/.

[gyaf068-B72] Rimbach R , Willigenburg R, Schoepf I, Yuen CH, Pillay N, Schradin C. 2016. Young but not old adult African Striped Mice reduce their activity in the dry season when food availability is low. Ethology 122(10):828–840. 10.1111/eth.12527

[gyaf068-B73] Scotcher JSB , Stewart DRM, Breen CM. 1978. The diet of the hippopotamus in Ndumu Game Reserve, Natal, as determined by faecal analysis. South African Journal of Wildlife Research 8:1–11. 10520/AJA03794369_3154

[gyaf068-B74] Skinner JD , Scorer JA, Millar RP. 1975. Observations on the reproductive physiological status of mature herd bulls, bachelor bulls, and young bulls in the hippopotamus *Hippopotamus amphibius amphibius* Linnaeus. General and Comparative Endocrinology 26(1):92–95. 10.1016/0016-6480(75)90218-X 1132668

[gyaf068-B75] van der Sluis T , Cassidy L, Brooks C, Wolski P, Vanderpost C, Wit P, Henkens R, Van Eupen M, Mosepele K, Maruapula O, et al 2017. Chobe district integrated land use plan. Wageningen (The Netherlands): Wageningen Environmental Research.

[gyaf068-B76] Stears K , McCauley DJ, Finlay JC, Mpemba J, Warrington IT, Mutayoba BM, Power ME, Dawson TE, Brashares JS. 2018. Effects of the hippopotamus on the chemistry and ecology of a changing watershed. Proceedings of the National Academy of Sciences 115(22):e5028–e5037. 10.1073/pnas.1800407115 PMC5984519 29760056

[gyaf068-B78] Stommel C , Hofer H, East ML. 2016. The effect of reduced water availability in the Great Ruaha River on the vulnerable common hippopotamus in the Ruaha National Park, Tanzania. PLoS ONE 11(6):e0157145. 10.1371/journal.pone.0157145 27276362 PMC4898818

[gyaf068-B79] Taylor R. 1976. Hippopotamuses at Lake St Lucia. In: Proceedings of St Lucia Scientific Advisory Council Workshop; 15-17 February 1976; Charter’s Creek (South Africa): Natal Parks Board, Pietrmaritzburg.

[gyaf068-B80] Timbuka CD. 2012. The ecology and behaviour of the common hippopotamus, *Hippopotamus amphibius* L. in Katavi National Park, Tanzania: responses to varying water resources [dissertation]. [Norwich (UK)]: University of East Anglia.

[gyaf068-B81] Tooth S , McCarthy TS. 2007. Wetlands in drylands: geomorphological and sedimentological characteristics, with emphasis on examples from southern Africa. Progress in Physical Geography 31(1):3–41. 10.1177/0309133307073879

[gyaf068-B82] UNESCO. 2014. Okavango Delta—UNESCO World Heritage Centre. [accessed 27 November 2016]. https://whc.unesco.org/en/news/1159/

[gyaf068-B83] Viljoen PC , Biggs HC. 1998. Population trends of hippopotami in the rivers of the Kruger National Park, South Africa. In: Dunstone N, Gorman Mm, editors. Behaviour and ecology of riparian mammals. Cambridge (UK): Cambridge University Press; p. 251–279.

[gyaf068-B85] Wilson RR , Jansen BD, Krausman PR. 2008. Planning and assessment of activity budget studies employing instantaneous sampling. Ethology 114(10):999–1005. 10.1111/j.1439-0310.2008.01544.x

[gyaf068-B86] Wolski P , Murray-Hudson M. 2006. Reconstruction 1989-2005 inundation history in the Okavango Delta from archival LandSat TM imagery. Globewetlands Symposium, ESA-ESRIN; 19 October 2006; Rome (Italy); p. 19–20.

[gyaf068-B87] Wolski P , Murray-Hudson M, Mazvimavi D, Ringrose S. 2008. Change and variability of flooding in the Okavango Delta, Botswana, and their consequences for water resources management. Second IASTED africa conference water resource management; 8 September 2008; Gaborone (Botswana): ACTA Press.

[gyaf068-B88] Wood S. 2017. Generalized additive models: an introduction with R. 2nd ed. New York (NY, USA): Chapman and Hall/CRC.

[gyaf068-B89] Young E. 1966. Nutrition of the hippopotamus (*Hippopotamus amphibius*). African Wildlife 20:165–167.

