# Supplementary material for: Sleeping giants: temporal, seasonal, and spatial variations in the 24-h activity budget of Hippopotamus amphibius
Source: J Mammal. 2025 Sep 19;106(6):1447–55. doi: 10.1093/jmammal/gyaf068 (PMC12854209; doi:10.1093/jmammal/gyaf068)
Supplement: gyaf068_Supplementary_Data [file gyaf068_supplementary_data.zip › SD1.pdf]

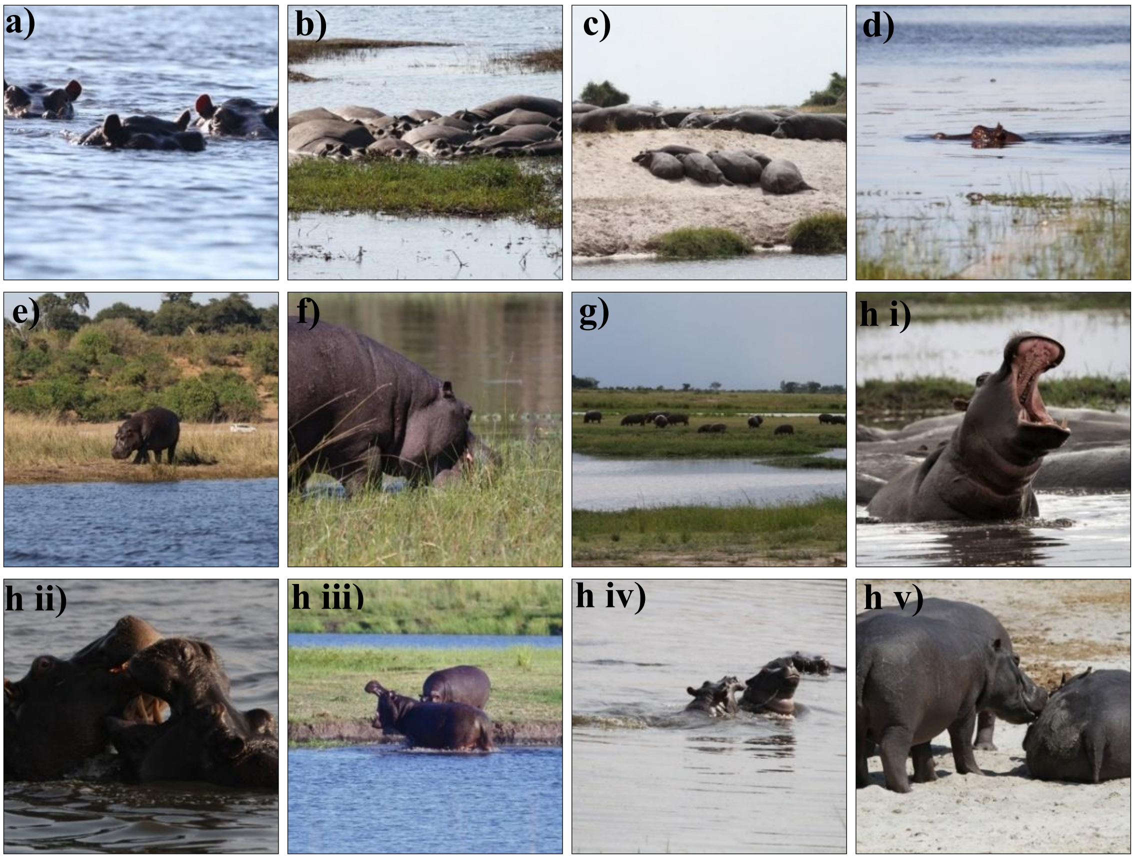

Supplementary Data SD1. Photo examples of a) resting deep aquatic (RDA), b) resting shallow aquatic (RSA), c) resting terrestrial (RT), d) moving aquatic (MA), e) moving terrestrial (MT), f) feeding aquatic (FA), g) feeding terrestrial (FT), and h) social (S): i) yawning ii) fighting, iii) aggression, iv) playing, v) grooming.
