# Supplementary material for: Sleeping giants: temporal, seasonal, and spatial variations in the 24-h activity budget of Hippopotamus amphibius
Source: J Mammal. 2025 Sep 19;106(6):1447–55. doi: 10.1093/jmammal/gyaf068 (PMC12854209; doi:10.1093/jmammal/gyaf068)
Supplement: gyaf068_Supplementary_Data [file gyaf068_supplementary_data.zip › SD2.pdf]

Supplementary Data SD2. Number of scans for each area for each season, for diurnal and nocturnal sessions.

Area	Diurnal			Nocturnal			Total
	**Dry (med-low)**	**Wet (low flood)**	**Dry (high flood)**	**Dry (med-low)**	**Wet (low flood)**	**Dry (high flood)**	
Chobe1	591	464	401	303	164	298	2221
Chobe2	896	474	393	246	257	322	2588
Chobe3	671	433	0	135	182	0	1421
Abu1	381	448	406	0	400	316	1951
**Total**	2539	1,819	1200	684	1003	936	
