# Supplementary material for: Sleeping giants: temporal, seasonal, and spatial variations in the 24-h activity budget of Hippopotamus amphibius
Source: J Mammal. 2025 Sep 19;106(6):1447–55. doi: 10.1093/jmammal/gyaf068 (PMC12854209; doi:10.1093/jmammal/gyaf068)
Supplement: gyaf068_Supplementary_Data [file gyaf068_supplementary_data.zip › SD3.pdf]

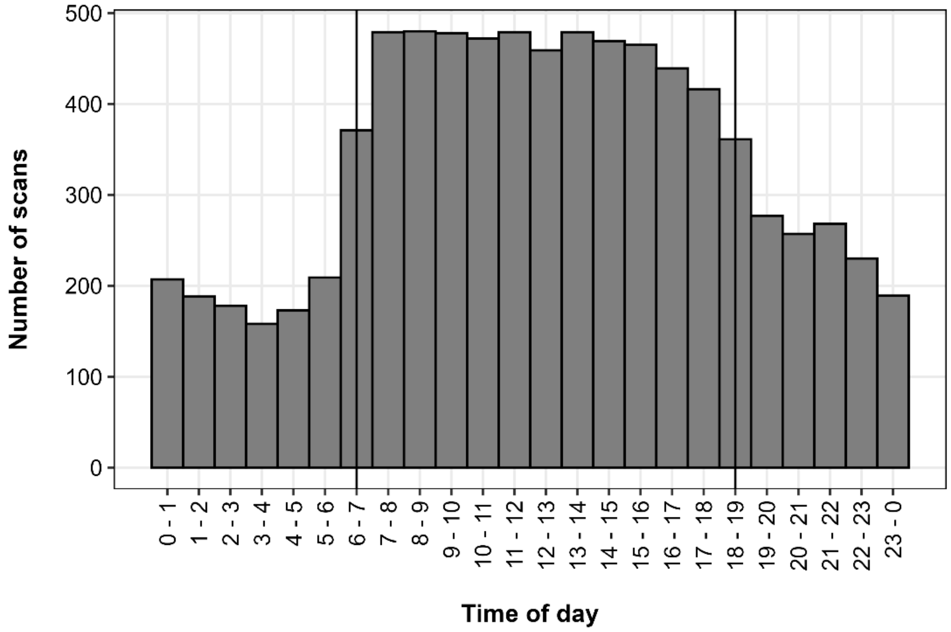

Supplementary Data SD3. Histogram of number of scans at different times of day. Vertical black lines denote approximate sunrise and sunset times.
