# Supplementary material for: Sleeping giants: temporal, seasonal, and spatial variations in the 24-h activity budget of Hippopotamus amphibius
Source: J Mammal. 2025 Sep 19;106(6):1447–55. doi: 10.1093/jmammal/gyaf068 (PMC12854209; doi:10.1093/jmammal/gyaf068)
Supplement: gyaf068_Supplementary_Data [file gyaf068_supplementary_data.zip › SD4.pdf]

Supplementary Data SD4. Mean, minimum, and maximum number of hippos per scans for each area for diurnal and nocturnal sessions.

Area	Time	Mean	Min	Max
Chobe1	Day	11	1	28
	Night	5	1	24
Chobe2	Day	23	1	110
	Night	7	1	41
Chobe3	Day	8	1	19
	Night	3	1	14
Abu1	Day	3	1	13
	Night	2	1	12
