# Supplementary material for: Sleeping giants: temporal, seasonal, and spatial variations in the 24-h activity budget of Hippopotamus amphibius
Source: J Mammal. 2025 Sep 19;106(6):1447–55. doi: 10.1093/jmammal/gyaf068 (PMC12854209; doi:10.1093/jmammal/gyaf068)
Supplement: gyaf068_Supplementary_Data [file gyaf068_supplementary_data.zip › SD5.pdf]

Supplementary Data SD5. Diurnal and nocturnal hippo activity budgets for study areas over three seasons (mean ± SE).

Area	Season	Time	RDA	RSA	RT	MA	MT	FA	FT	S
Chobe1	Dry season (high flood)	Day	37.2 ± 3.1	31.9 ± 4.1	2.6 ± 1.0	23.2 ± 3.2	0.3 ± 0.2	1.2 ± 0.5	1.8 ± 1.1	1.8 ± 0.5
		Night	38.3 ± 5.3	13.3 ± 3.6	6.1 ± 4.0	21.4 ± 2.9	3.1 ± 1.2	0.6 ± 0.4	15.2 ± 4.5	1.9 ± 0.6
	Dry season (med-low flood)	Day	54.5 ± 3.9	4.5 ± 1.8	16.4 ± 3.9	18.5 ± 2.2	1.3 ± 0.3	0.2 ± 0.1	1.6 ± 0.7	3.0 ± 0.7
		Night	33.7 ± 5.8	1.0 ± 0.3	18.6 ± 6.0	17.2 ± 4.1	3.5 ± 1.1	0.0 ± 0.0	24.0 ± 6.2	2.0 ± 1.0
	Wet season (low flood)	Day	6.4 ± 1.9	74.8 ± 4.9	0.5 ± 0.2	9.0 ± 1.9	0.4 ± 0.1	0.3 ± 0.1	6.7 ± 1.9	1.9 ± 0.7
		Night	29.4 ± 7.5	12.3 ± 6.6	0.8 ± 0.5	18.6 ± 4.9	3.7 ± 1.9	0.8 ± 0.6	31.9 ± 9.6	2.3 ± 1.3
Chobe2	Dry season (high flood)	Day	51.4 ± 2.6	11.6 ± 2.6	0.0 ± 0.0	34.6 ± 3.2	0.0 ± 0.0	1.0 ± 0.4	0.0 ± 0.0	1.3 ± 0.3
		Night	40.2 ± 3.9	21.3 ± 3.0	8.9 ± 3.2	23.4 ± 2.3	1.9 ± 0.7	1.0 ± 0.9	2.7 ± 1.4	0.6 ± 0.3
	Dry season (med-low flood)	Day	18.3 ± 2.4	57.7 ± 3.5	1.0 ± 0.3	8.0 ± 0.9	1.2 ± 0.2	0.4 ± 0.1	12.0 ± 1.9	1.4 ± 0.3
		Night	19.5 ± 5.3	2.5 ± 1.6	47 ± 8.5	6.9 ± 2.0	2.9 ± 0.9	0.0 ± 0.0	19.3 ± 5.6	1.8 ± 0.5
	Wet season (low flood)	Day	34.0 ± 4.2	31.1 ± 5.4	0.9 ± 0.3	11.9 ± 1.5	1.1 ± 0.2	0.0 ± 0.0	19.5 ± 3.6	1.5 ± 0.3
		Night	37.1 ± 5.7	17.5 ± 6.6	0.0 ± 0.0	14.6 ± 2.0	1.4 ± 0.5	0.0 ± 0.0	24.2 ± 7.3	5.2 ± 1.8
Chobe3	Dry season (high flood)									
	Dry season (med-low flood)	Day	58.3 ± 2.4	13.7 ± 2.0	5.8 ± 2.2	19.8 ± 1.9	0.2 ± 0.1	0.1 ± 0.1	0.2 ± 0.2	1.8 ± 0.5
		Night	39.6 ± 8.8	0.9 ± 0.6	2.3 ± 1.5	12.1 ± 3.2	2.2 ± 1.2	0.0 ± 0.0	40.7 ± 10.	2.2 ± 1.7
	Wet season (low flood)	Day	57.1 ± 3.1	21.2 ± 4.3	0.0 ± 0.0	19.9 ± 2.0	0.0 ± 0.0	0.0 ± 0.0	0.0 ± 0.0	1.8 ± 0.5
		Night	49.4 ± 7.1	4.5 ± 1.5	0.0 ± 0.0	12.6 ± 3.0	2.8 ± 1.8	0.6 ± 0.6	29.5 ± 8.6	0.5 ± 0.3
Abu1	Dry season (high flood)	Day	45.9 ± 3.4	22.7 ± 3.9	0.5 ± 0.4	26.1 ± 2.8	0.1 ± 0.1	2.5 ± 1.3	0.2 ± 0.1	1.9 ± 0.5
		Night	33.5 ± 4.8	12.2 ± 2.4	0.9 ± 0.4	19.5 ± 3.2	0.7 ± 0.3	27.2 ± 5.2	4.3 ± 1.8	1.7 ± 1.1
	Dry season (med-low flood)	Day	8.2 ± 2.2	60.3 ± 7.2	7.8 ± 4.1	16.1 ± 4.1	0.5 ± 0.3	0.0 ± 0.0	0.1 ± 0.1	7.1 ± 2.8
	Wet season (low flood)	Day	54.2 ± 4.6	23.3 ± 4.6	0.0 ± 0.0	15.1 ± 3.1	0.1 ± 0.1	0.0 ± 0.0	0.2 ± 0.2	7.2 ± 2.3
		Night	19.6 ± 4.8	21.3 ± 5.2	0.2 ± 0.2	6.2 ± 2.1	5.2 ± 2.0	0.0 ± 0.0	39.0 ± 6.3	8.5 ± 3.3
