# Supplementary material for: Sleeping giants: temporal, seasonal, and spatial variations in the 24-h activity budget of Hippopotamus amphibius
Source: J Mammal. 2025 Sep 19;106(6):1447–55. doi: 10.1093/jmammal/gyaf068 (PMC12854209; doi:10.1093/jmammal/gyaf068)
Supplement: gyaf068_Supplementary_Data [file gyaf068_supplementary_data.zip › SD6.pdf]

Supplementary Data SD6 - Notable field observations

Resting terrestrial and sun exposure

The longest continuous bout of resting terrestrial was by three hippos at Chobe2 for six hours (21:30–03:30, September 2017). Another notable occurrence occurred in October 2017, where 24 hippos rested terrestrial as a group for 3.5 hours (22:35–02:05, Chobe1). Diurnally, the longest uninterrupted bout was by one juvenile, who rested terrestrial for almost three hours from 09:15 to 12:25 (May 2018, Chobe1). In September 2018, two hippos rested terrestrial for almost 2.5 hours (14:10–16:30, Chobe3) with an average temperature during this time of 35.5°C. In addition, there were multiple occurrences of most hippos in Chobe1 (up to 28 hippos) hauling out from the water and resting terrestrial on the sandbank for around 2.5 hours in the middle of the day. These values are the minima; there were often hippos resting terrestrial before/after the given times, but we could rarely identify individual hippos.

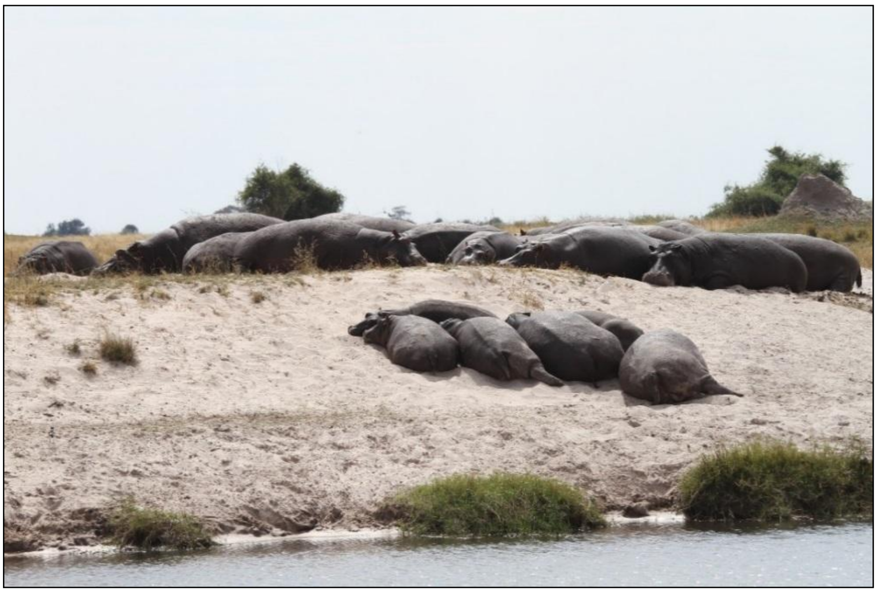

Chobe1 hippo pod resting terrestrial on the sandbank adjacent to their lagoon.

We did not observe hippo skin cracking and found only two examples in the literature where cracking of skin had been directly observed: a hippo kept out of water for six days (Arman &

Field, 1973) and a juvenile zoo animal (Young, 1966). A subsequent study showed that a hippo denied access to water to cool on a hot day was able to effectively thermoregulate through controlled EWL, with no obvious consequences (no adverse skin effects) and no apparent discomfort (it remained placid throughout the nine hours) (Wright, 1975, 1987).

Feeding aquatic

Hippos were observed feeding on several species of aquatic vegetation, primarily grass species, but also on blue water lily (*Nymphaea nouchali*) and the stem of papyrus (*Cyperus papyrus*). In other areas, hippos have been recorded feeding on a variety of aquatic plants, including bulrush, Nile cabbage, water lilies, submerged macrophytes, aquatic grasses, sedges, and reeds—sometimes in large quantities (Taylor, 1975; Hoven, 1978; Mugangu & Hunter, 1992; Harrison et al., 2008; Klingel, 2013; Mekonen & Hailemariam, 2016; Prinsloo, 2016). However, they are still widely referred to as exclusively terrestrial consumers. Feeding on aquatic vegetation may occur in response to the scarcity or lower nutritional quality of terrestrial grasses, which have lower crude protein levels, particularly in the dry season (Mugangu & Hunter, 1992; Harrison et al., 2008; Klingel, 2013; Prinsloo, 2016). Alternatively, it may simply reflect the availability of palatable aquatic vegetation due to seasonal flooding (Harrison et al., 2008; Klingel, 2013).

Unlike their terrestrial feeding style—where grasses are plucked with their lips while their snout faces downward—hippos feeding on aquatic vegetation use a different method. They take plants into their mouth, chew to break the stems, and throw their head back to swallow. This method is particularly slow when consuming papyrus and water lilies, suggesting that aquatic feeding may be less efficient than terrestrial grazing, potentially explaining why hippos do not always utilize this resource (Mugangu & Hunter, 1992). Diet flexibility in hippos has been

noted in previous studies, with reports of them feeding on overhanging terrestrial plants when available (Taylor, 1975, Mugangu & Hunter, 1992). This adaptability indicates that hippo diet is influenced by habitat conditions and plant availability.

Interactions with predators, other animals, and humans

We observed several interactions between hippos and predators, but never a successful attack or injuring. In August 2017, hippos at Chobe1 were resting terrestrial as a group when three lionesses approached and one pounced on a juvenile. Before the attack, the hippos were not vigilant, appearing not to notice the approaching lions although they were moving through an open area. Upon attack, all the hippos (including the juvenile) ran into the water and watched the lions move past the lagoon. The juvenile did not appear to be injured. After approximately 30 minutes, the hippos begun to move back onto land, although several times they became disturbed by unknown sources and ran back into the water, before again moving out. Towards the middle of the day the hippos settled and rested terrestrial without interruption.

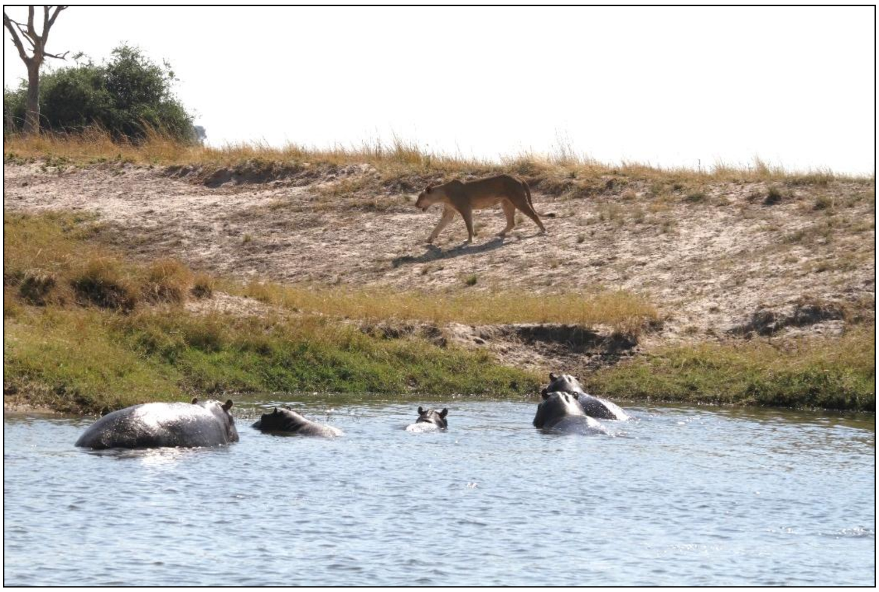

Hippos at Chobe1 watching a lion move past after an attempted attack on a juvenile.

On another occasion, a lion walked past hippos resting aquatic, but the hippos did not appear to be disturbed. Another interaction occurred when a lone adult bull hippo at Abu1 left the water at sunset and begun feeding when a leopard ran towards the hippo from behind. The hippo turned to face the leopard without any other apparent aggressive display, and the leopard retreated. Several times, leopards and wild dogs were seen drinking from the water where hippos were resting, and the hippos remained vigilant but did not react in any other way. Hippos rarely seemed disturbed by hyena or jackal. We often observed hippos close to large crocodiles both in the water and on land, without any apparent changes in behaviour (though see Inman & Leggett (2019)). Hippos were often disturbed by elephants moving through the water, normally reacting passively by vocalising, yawning and moving away, although on several occasions we observed hippos acting aggressively, by moving towards the elephants and blocking their path, but retreating when the elephants charged. Hippos that were resting on land were sometimes disturbed by elephants walking nearby and would retreat into the water. No physical contact was ever observed between the two species. Clarke (1953) also noted that hippos were disturbed by elephants.

The hippos were often vigilant towards, and disturbed by, the presence of humans, particularly when on land. Hippos acted aggressively towards me twice, one instance is mentioned in Inman & Leggett (2019), and the other occurred after the above-mentioned leopard incident, where the hippo charged my car, stopping after a short distance. Disturbances were often accompanied by group vocalisations and almost always with all hippos retreating to the water. A single hippo entering the water would often result in all other hippos rushing back to the water, even if there appeared to be no obvious catalyst. This sensitivity to human disturbance has been noted elsewhere. During boat surveys, hippos lying on sandbanks rushed into the water upon seeing the observers (Ansell 1965), hippos preferring to bask in areas where human disturbance is minimal (Clarke, 1953; Klingel, 1991a, 2013; Onyeanusi, 2004), and hippos leaving the water

at night for feeding often avoid people (Onyeanusi, 2004; Prinsloo, 2016). Hippos that had previously rested on land stopped this activity following a period of strong disturbance (e.g. hunting, persecution) (Ansell, 1965; Klingel, 1991a; Bouché, 2004a). Preferentially resting terrestrial close to water (personal observation; Eltringham, 1999; Nuñez, 2017) has been explained as allowing a quick return in case of overheating (Eltringham 1999), but could also represent attempts to remain close to water for safety. Hippos undoubtedly feel vulnerable on land (Eltringham, 1999; Bouché, 2004a; Onyeanusi, 2004).

Social and other activities

Mating was observed seven times and in all three seasons; once on 18 January 2018 (Chobe3, diurnal), twice on 21 May 2018 (Chobe1, diurnal), once on 29 May 2018 (Chobe1, nocturnal), and three times on 8 September 2018 (Chobe2, diurnal). Consistent with other studies, we observed hippos mating more than once in a day (Mekonen & Hailemariam, 2016; Klingel, 2013; Krueger, 1997), but we could not confirm if it was the same bull and female mating. We only observed mating occurring in water, consistent with other studies (Laws et al., 1966; Scotcher, 1973; Klingel, 1991b, 2013; Eltringham, 1999; but see Onyeanusi, 2004), with the female almost entirely submerged, raising her nostrils to breathe every several minutes. Other hippos, particularly juveniles and subadults, would often remain close, diving around the mating pair, confirmed by Ansell (1965). We never observed the copulating pair displaying aggressive behaviour, which has been seen elsewhere (Scotcher, 1973; Krueger, 1997).

Grooming (licking) of conspecifics was a rare occurrence, recorded three times. Dung paddling occurred often, only ever by adult males, normally immediately when a hippo left or entered the water, but also when hippos were on land, sometimes far from the water. At Abu1, we observed two bulls leave the water and walk together, both stopping several times to dung

paddle on the same spots one directly after the other. Despite the known territoriality of hippos, we only observed fighting between bulls three times (once, September 2017; twice, January 2018), though non-serious sparring was a common occurrence, especially by two bulls in Abu1.

The activity category 'other' was discarded prior to analysis due to the low number of times recorded (approximately 0.04% of the scans). Behaviours that were recorded as 'other' included rolling in the water, geophagia of sand (once, September 2017), blowing bubbles (differed from bubbles produced during communication), drinking (twice, September 2017 and February 2018), nursing (once, January 2018), and scratching snout on the sand. Nursing occurs predominately in water, with the female lying on her side and the juvenile submerging to suckle (Laws & Clough, 1966), which may be why it was so rarely observed.

References

Ansell, W.F.H. (1965). Hippo census on the Luangwa River. *The Puku*. 32 (3). 647–655.

Arman, P. & Field, C.R. (1973). Digestion in the hippopotamus. *African Journal of Ecology*. 11 (1). 9–17.

Bouché, P.J.C. (2004a). *Ecology and activity of hippopotamuses in the White Bandama River, Ivory Coast*. [Online]. Available from: https://www.researchgate.net/publication/311921686_Ecology_and_activity_of_Hippopotamus_in_the_White-Bandama_River_Ivory_Coast.

Clarke, J.R. (1953). The hippopotamus in Gambia, West Africa. *Journal of Mammalogy*. 34 (3). 299–315.

Eltringham, S.K. (1999). *The hippos: natural history and conservation*. London: Academic Press.

Harrison, M.E., Kalindekafe, M.P. & Banda, B. (2008). The ecology of the hippopotamus in Liwonde National Park, Malawi: Implications for management. *African Journal of Ecology*. 46 (4). 507–514.

Hoven, W.V.A.N. (1978). Digestion physiology in the stomach complex and hindgut of the hippopotamus (*Hippopotamus amphibius*). *South African Journal of Wildlife Research*. 8. 59–64.

Klingel, H. (1991a). Sizing up a heavyweight. *International Wildlife*. 21 (5). 4

Klingel, H. (2013). *Hippopotamus amphibius* Common Hippopotamus. In: J. Kingdon & M. Hoffman (eds.). *Mammals of Africa: Volume VI: Pigs, Hippopotamuses, Chevrotain, Giraffes, Deer and Bovids*. London, UK: Bloomsbury Publishing, 68–77.

Krueger, S. (1997). Hippopotamus underwater behavior and communication. *Animal Keepers Forum*. 24 (3). 108–110.

Laws, R.M. & Clough, G. (1966). Observations on reproduction in the hippopotamus *Hippopotamus amphibius* Linn. In: I. W. Rowlands (ed.). *Comparative Biology of*

Reproduction in Mammals. London, UK: Academic Press, 117–140.

Mekonen, S. & Hailemariam, B. (2016). Ecological behaviour of common hippopotamus (*Hippopotamus amphibius*, LINNAEUS, 1758) in Boye wetland, Jimma, Ethiopia. *American Journal of Scientific and Industrial Research*. 7 (2). 41–49.

Mugangu, T.E. & Hunter, M.L.J. (1992). Aquatic foraging by Hippopotamus in Zaïre: response to a food shortage? *Mammalia*. 56 (3). 345–349.

Nuñez, T.A. (2017). *Animal movement in a changing world*. PhD Thesis. University of California.

Onyeanusi, A.E. (2004). Some behavioural characteristics of common hippopotamus (*H. amphibius* Linn. 1758) in Nigeria's Kainji Lake National Park. *International Journal of Agriculture and Rural Development*. 5 (1). 27–35.

Scotcher, J.S.B., Stewart, D.R.M. & Breen, C.M. (1978). The diet of the hippopotamus in Ndumu Game Reserve, Natal, as determined by faecal analysis. *South African Journal of Wildlife Research*. 8. 1–11.

Taylor, R. (1975). *Hippopotamuses at Lake St. Lucia*.

Wright, P.G. (1975). Thermoregulation on land, and the hippopotamus. *South African Medical Journal*. 49. 275.

Wright, P.G. (1987). Thermoregulation in the hippopotamus on land. *South African Journal of Zoology*. 22 (3). 237–242.

Young, E. (1966). Nutrition of the hippopotamus (*Hippopotamus amphibius*). *African Wildlife*. 20. 165–167.
