# Supplementary material for: Sleeping giants: temporal, seasonal, and spatial variations in the 24-h activity budget of Hippopotamus amphibius
Source: J Mammal. 2025 Sep 19;106(6):1447–55. doi: 10.1093/jmammal/gyaf068 (PMC12854209; doi:10.1093/jmammal/gyaf068)
Supplement: gyaf068_Supplementary_Data [file gyaf068_supplementary_data.zip › SD7_14.docx]

**Supplementary Data SD7.** Summary of RDA GAM model

**
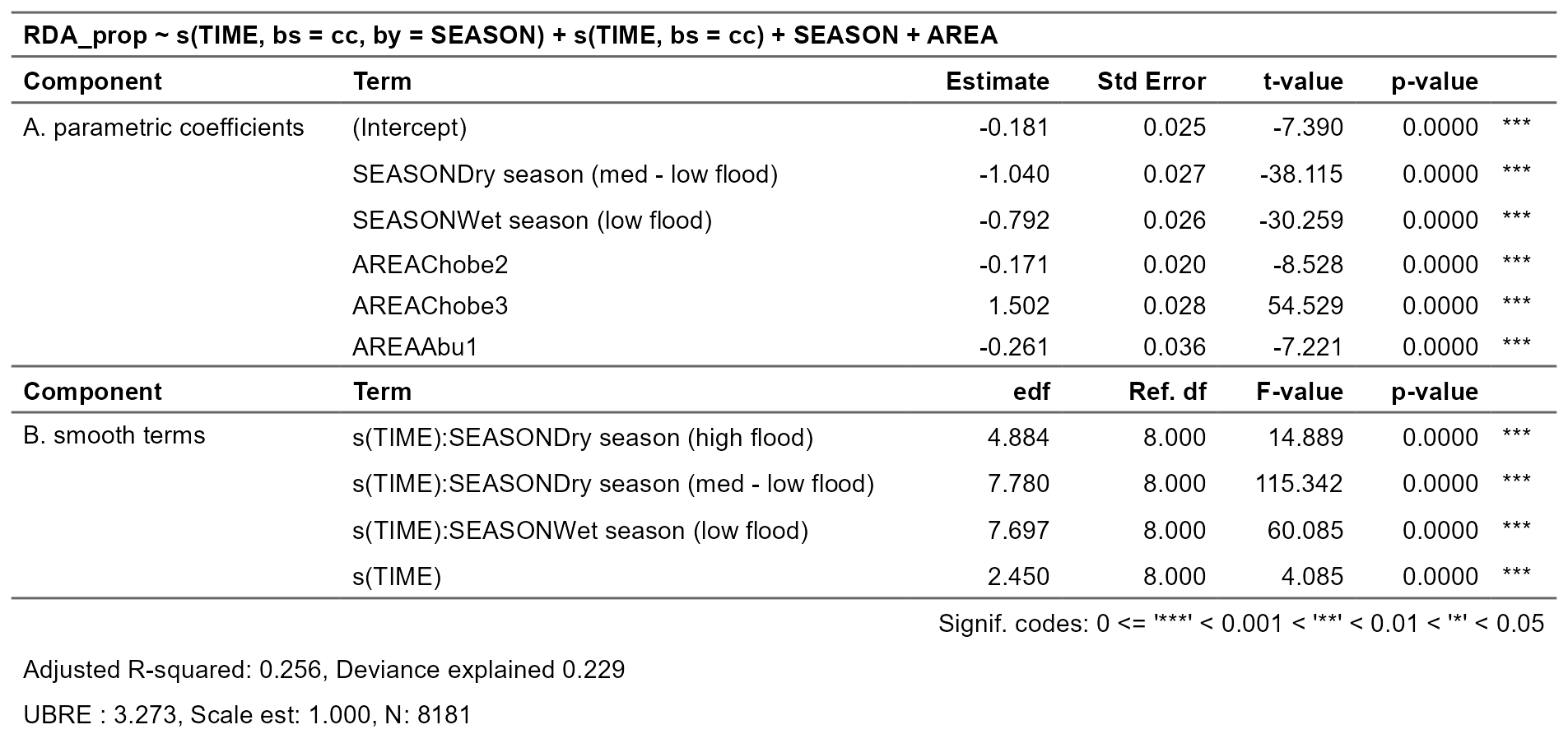
**

**Supplementary Data SD8.** Summary of RSA GAM model

**
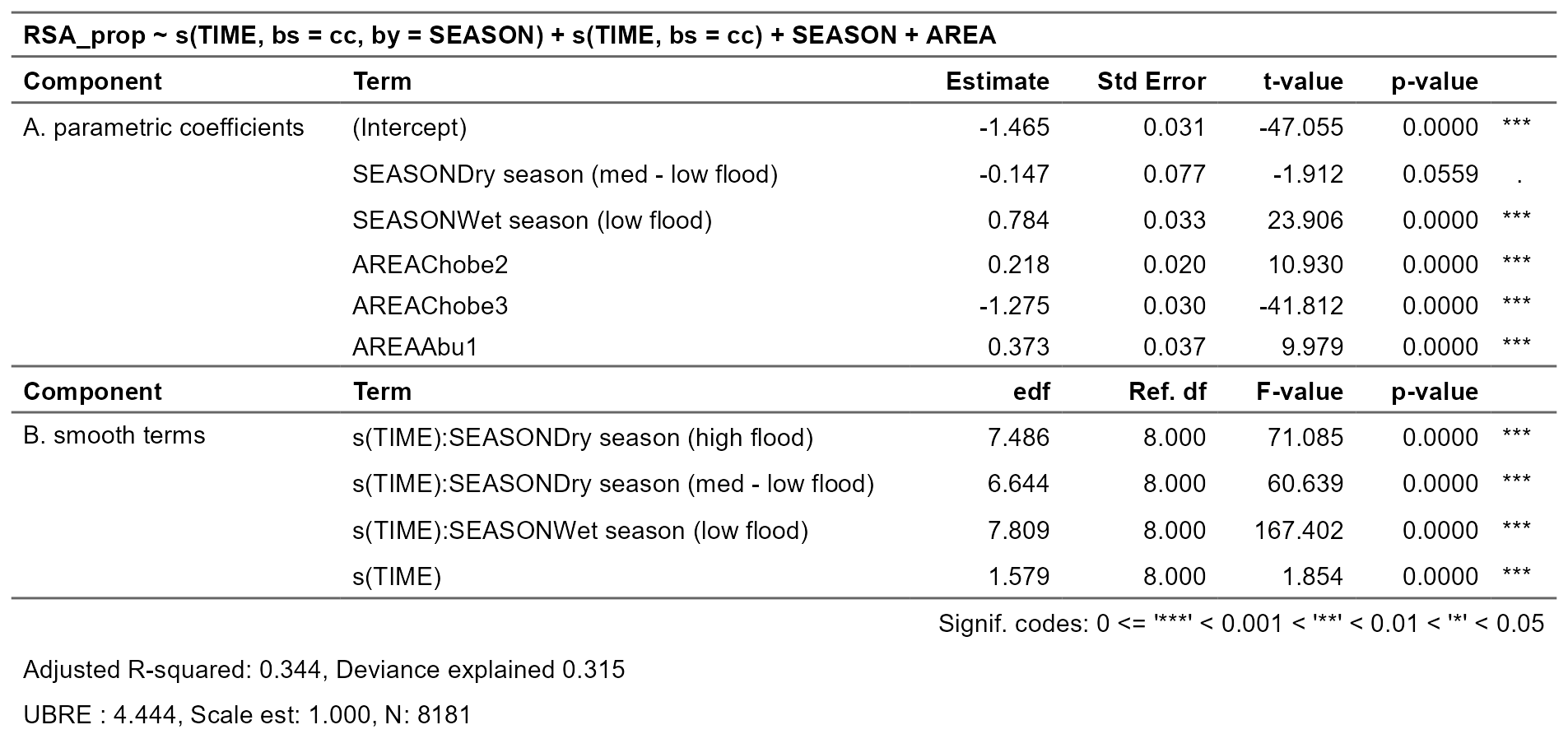
**

**Supplementary Data SD9.** Summary of RT GAM model

**
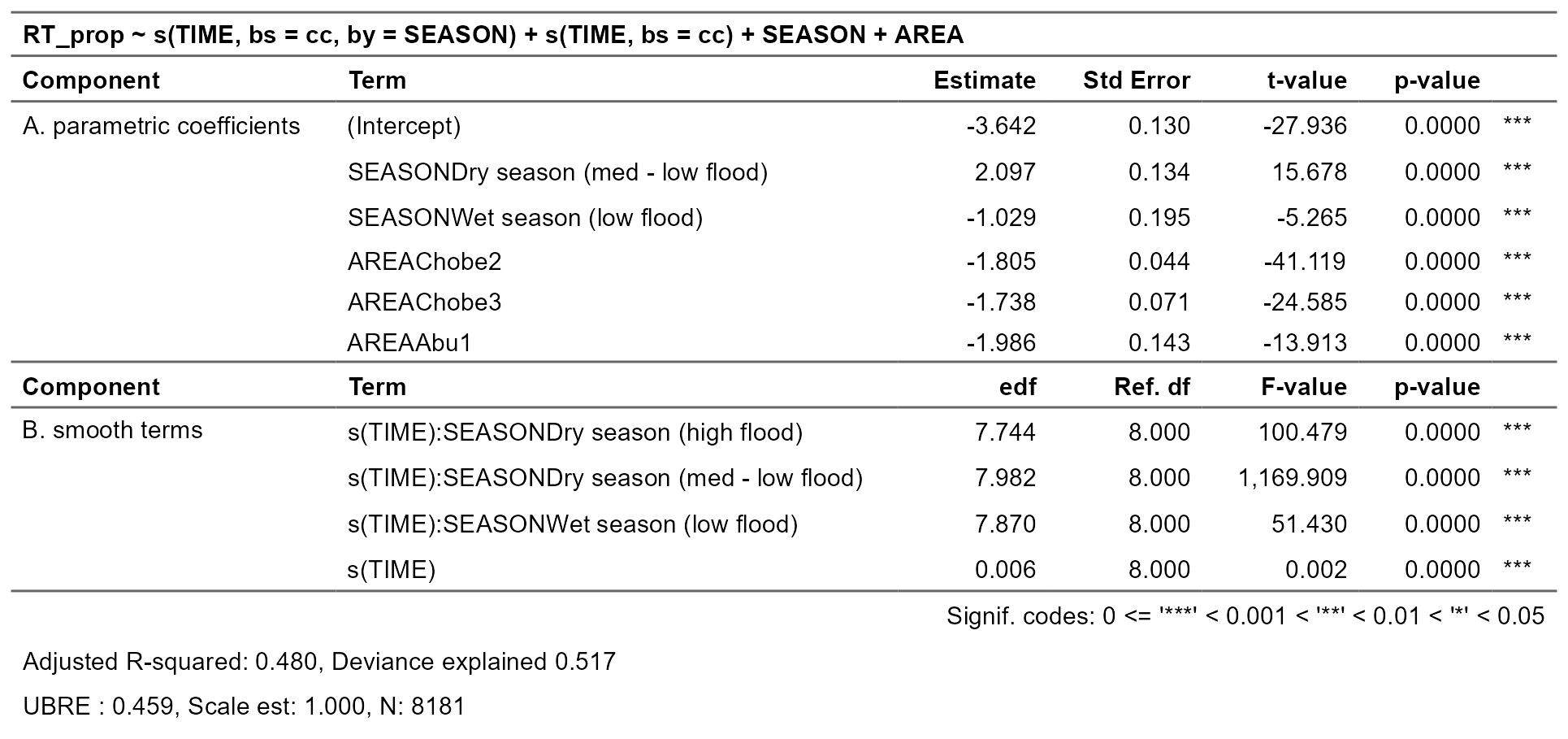
**

**Supplementary Data SD10.** Summary of MA GAM model

**
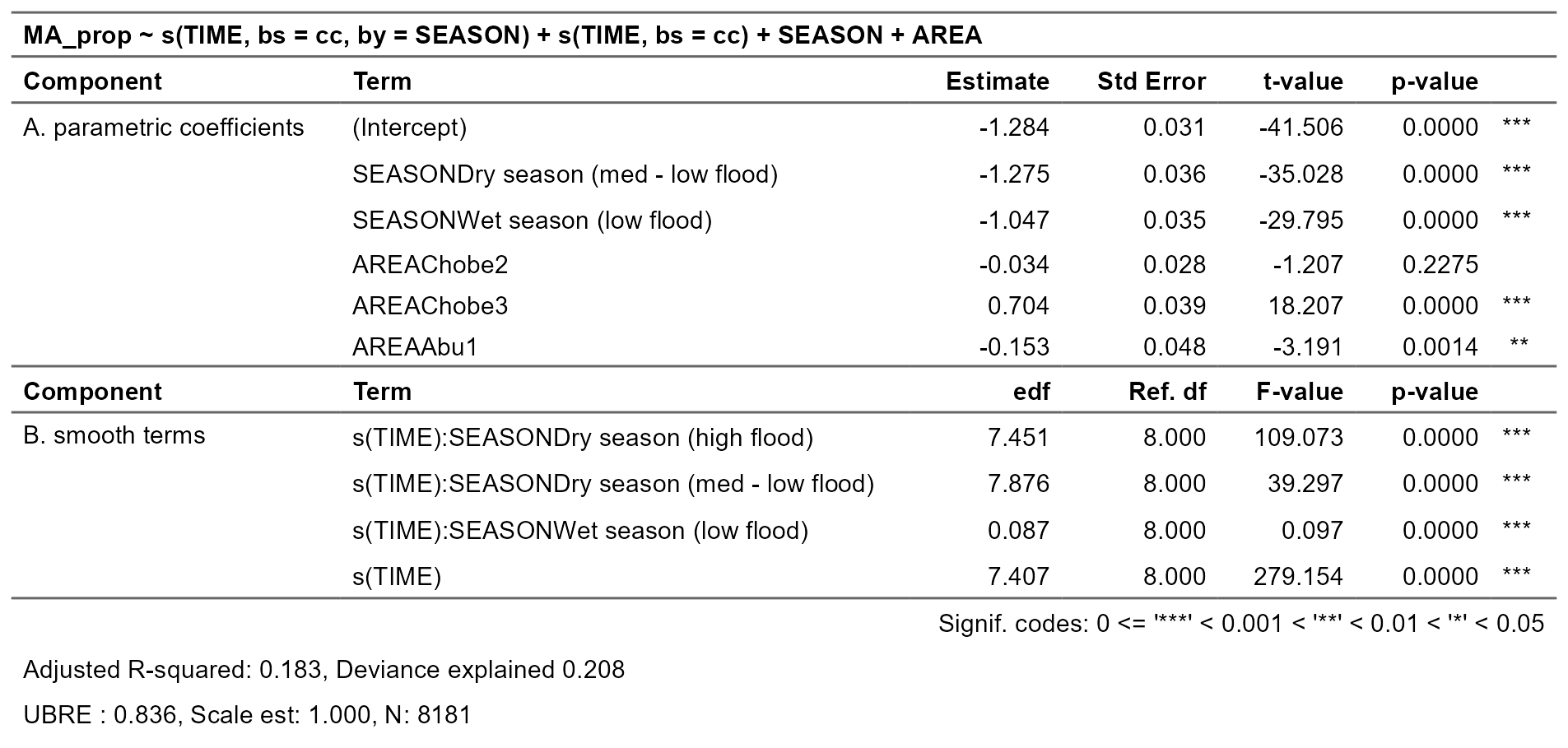
**

**Supplementary Data SD11.** Summary of MT GAM model

**
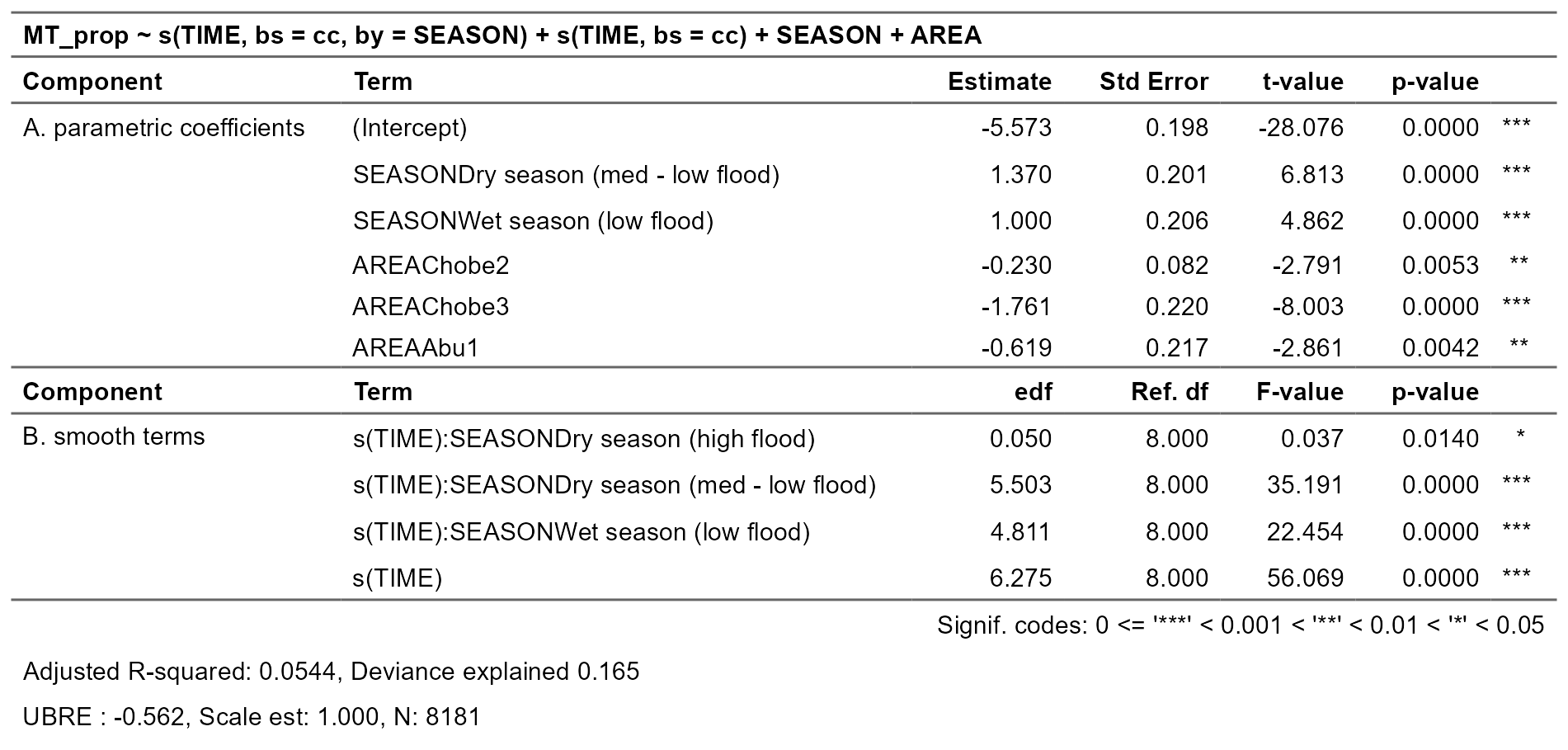
**

**Supplementary Data SD12.** Summary of FA GAM model

**
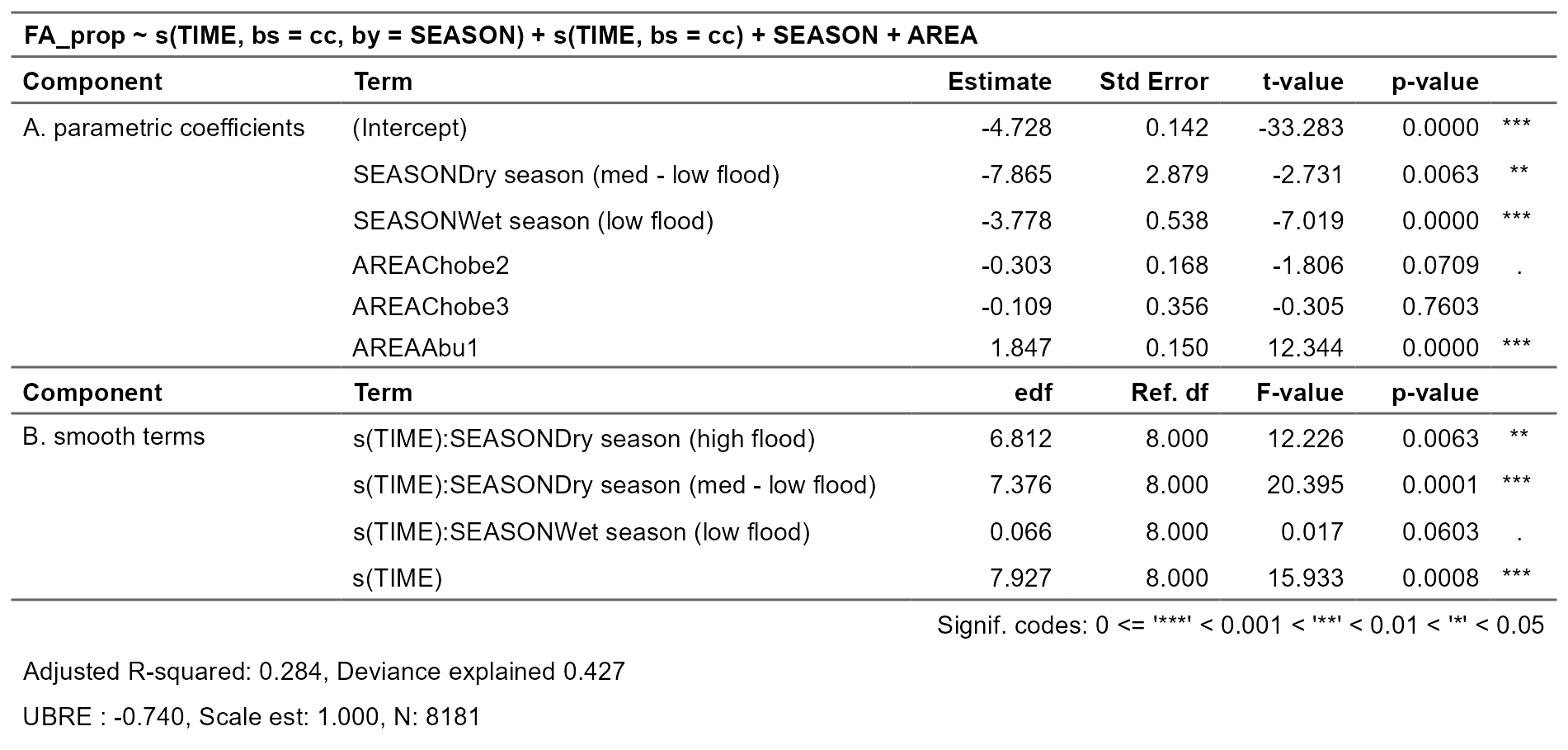
**

**Supplementary Data SD13.** Summary of FT GAM model

**
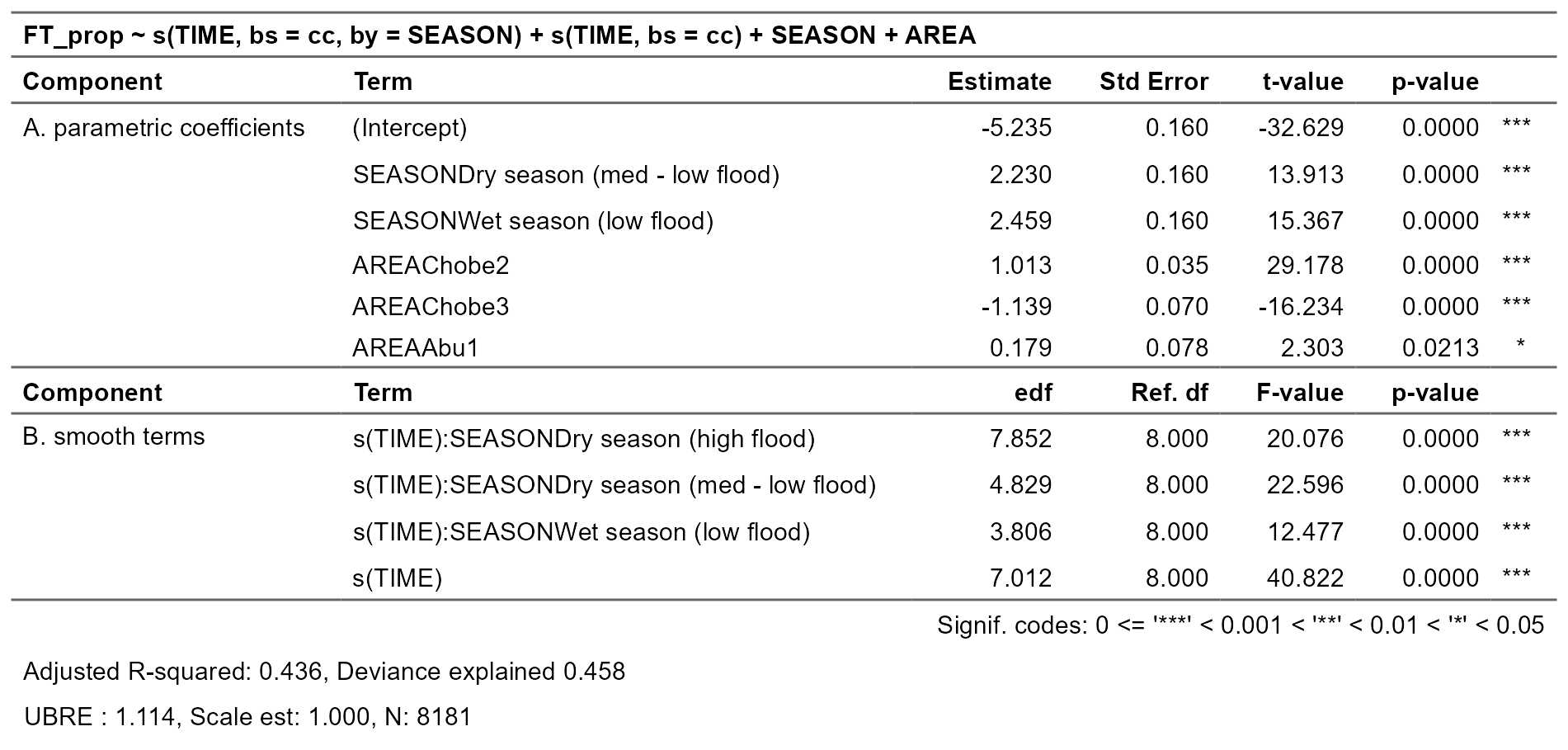
**

**Supplementary Data SD14.** Summary of S GAM model

**
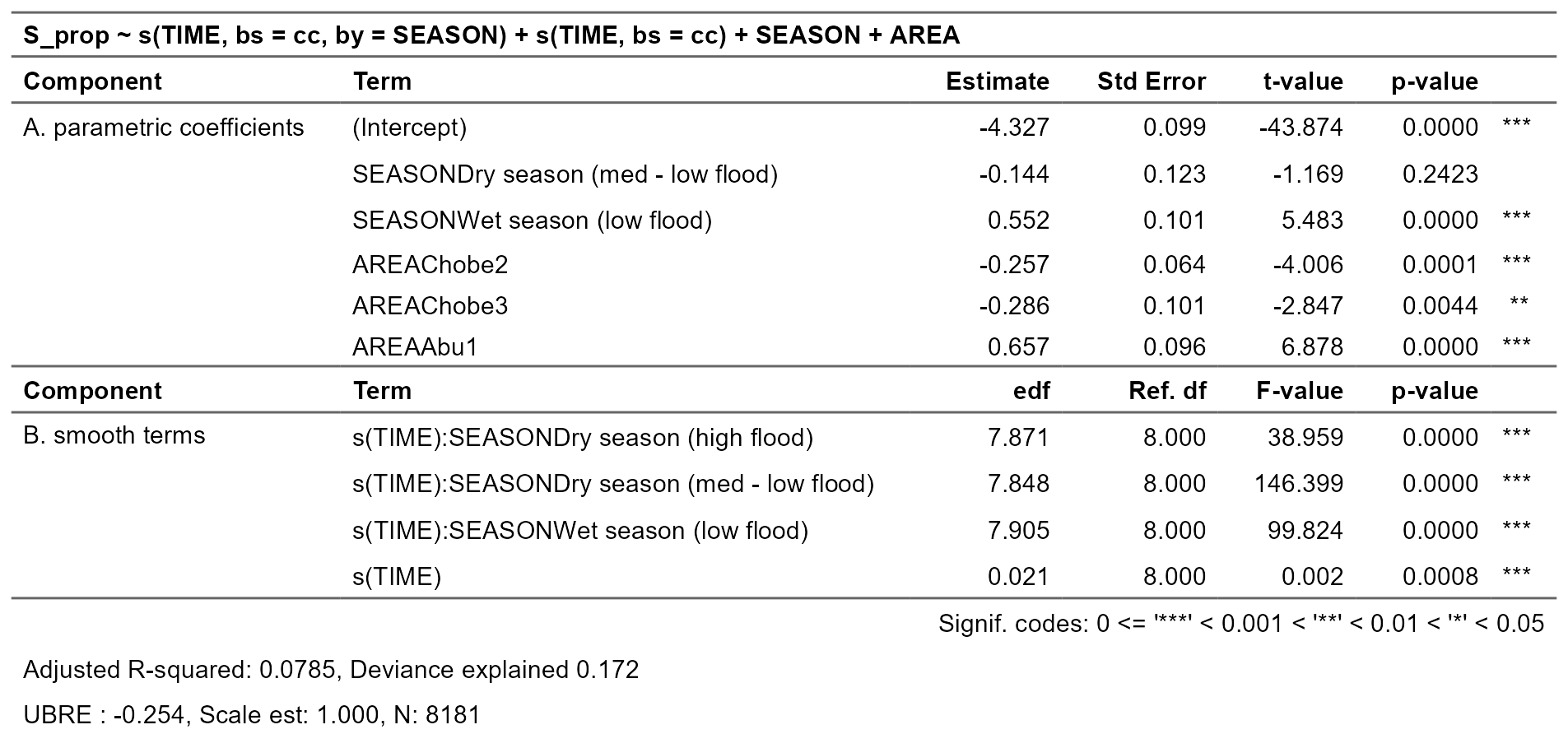
**
